# Liquid Printing in Nanochitin Suspensions: Interfacial Nanoparticle Assembly Toward Volumetric Elements, Organic Electronics and Core–Shell Filaments

**DOI:** 10.1002/smtd.202500100

**Published:** 2025-03-03

**Authors:** Mahyar Panahi‐Sarmad, Ahmadreza Ghaffarkhah, Lukas Alexander Bauman, Amin Babaei‐Ghazvini, Seyyed Alireza Hashemi, Bishnu Acharya, Boxin Zhao, Mohammad Arjmand, Feng Jiang, Orlando J Rojas

**Affiliations:** ^1^ Department of Wood Science The University of British Columbia 2424 Main Mall #2900 Vancouver BC V6T 1Z1 Canada; ^2^ Bioproducts Institute University of British Columbia 2385 East Mall Vancouver BC V6T 1Z4 Canada; ^3^ Department of Chemical and Biological Engineering University of British Columbia 2360 East Mall Vancouver BC V6T 1Z3 Canada; ^4^ Nanomaterials and Polymer Nanocomposites Laboratory School of Engineering University of British Columbia Kelowna BC V1 V 1V7 Canada; ^5^ Department of Chemical Engineering University of Waterloo 200 University Avenue West Waterloo ON N2L 3G1 Canada; ^6^ Department of Chemical and Biological Engineering University of Saskatchewan 57 Campus Drive Saskatoon SK S7N5A9 Canada; ^7^ Department of Chemistry The University of British Columbia 2036 Main Mall Vancouver BC V6T 1Z1 Canada

**Keywords:** core–shell filaments, high‐resolution extrusion, liquid‐in‐liquid printing, nanoparticle interfacial assembly, partially miscible interfaces

## Abstract

A nanoparticle‐nanoparticle assembly is introduced using electrostatic complexation to precisely control volumetric structuring at the water/alcohol interface. In this system, an aqueous graphene oxide (GO) ink interacts electrostatically with partially deacetylated chitin nanofibers (mChNF), modified with benzophenone and dispersed in 1‐butanol, which serves as the external phase. Upon extrusion of the GO ink, a jammed interfacial network forms, stabilizing the printed patterns within the external suspension, which provides suitable viscoelasticity for support‐free printing. This approach is further extended to inks incorporating metal‐organic frameworks or cellulose nanoparticles, demonstrating the advantages of mChNF as a stabilizer. Additionally, by incorporating a conductive polymer, the inks can be tailored for programmable and conductive patterning, opening new opportunities in liquid electronics and reconfigurable systems. Finally, GO inks containing an anionic polyelectrolyte (sodium alginate) undergo osmosis‐driven solidification, facilitating the demolding of high‐fidelity 3D structures formed by the printed threads of struts. These structures exhibit coreshell morphologies and high mechanical strength (∼175 MPa at 4% strain). Overall, this liquid‐in‐liquid fabrication approach, enabled by the integration of mChNF in the external phase, unlocks new possibilities for the design of versatile and multifunctional materials.

## Graphical abstract

This study introduces liquid‐in‐liquid printing using chitin nanofibers (mChNF) that stabilize high‐fidelity structures by electrostatic complexation at the water/1‐butanol interface. Aqueous dispersions of graphene‐oxide (ink) and mChNF/1‐butanol (external phase) undergo nanoparticle jamming under rheological control with mChNF, facilitating the synthesis of volumetric structures in liquid media. This fabrication approach supports hybrid inks, yielding functional materials, core–shell morphologies, and soft electronics.

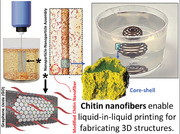

## 1 Introduction

Interfacially driven liquid‐in‐liquid printing is a transformative approach to additive manufacturing that enables an aqueous flowable stream to be shaped within an external phase.^[^ ^1^, ^2^, ^3^ ^]^ Unlike conventional printing methods that rely on high‐viscosity inks to achieve structure, liquid‐in‐liquid printing leverages the interfacial interactions between nanoparticle‐surfactant or nanoparticle‐nanoparticle jamming at the interface,^[^ ^3^, ^4^, ^5^ ^]^ resulting in non‐equilibrium, kinetically stable structures.^[^ ^4^, ^5^, ^6^, ^7^ ^]^ This jamming occurs when nanoparticles in one liquid phase bind ligands or complementary nanoparticles in the other phase, pinning an interface that counteracts Rayleigh instabilities.^[^ ^8^, ^9^, ^10^, ^11^, ^12^ ^]^ This effect works against coalescence, enabling the formation of structures without the need for high‐viscosity or viscoelastic materials.^[^ ^13^, ^14^, ^15^ ^]^ These structured fluids can be shaped into prescribed patterns.^[^ ^16^, ^17^, ^18^, ^19^ ^]^

Liquid‐in‐liquid printing presents several benefits over traditional additive manufacturing techniques that can undergo nozzle clogging, depend on the viscoelasticity of the ink^[^ ^20^, ^21^, ^22^ ^],^ and require supporting scaffolds.^[^ ^23^, ^24^ ^]^ The chemistry of nanoparticle‐surfactant or nanoparticle‐nanoparticle interactions can be tailored, allowing for precise control over the fabrication process, printed scale, and functionality,^[^ ^25^, ^26^, ^27^ ^]^ leading to applications such as microfluidics, soft robotics, and liquid electronics.^[^ ^28^, ^29^, ^30^ ^]^ Despite the potential of liquid‐in‐liquid printing, significant challenges persist, particularly in achieving high printing resolution and ensuring the stability of 3D‐printed structures.^[^ ^31^ ^]^ These issues can be addressed through effective interfacial complexation and careful optimization of the rheological properties of the external phase.^[^ ^32^, ^33^ ^]^ In essence, the external phase must possess appropriate viscoelastic characteristics to support printed patterns and prevent deformation during extrusion.^[^ ^34^, ^35^ ^]^ Without sufficient interfacial jamming and rheological tuning, printed structures are prone to collapse or spreading.

Current liquid‐in‐liquid printing methods predominantly rely on immiscible water/oil systems and highly viscous inks.^[^ ^36^, ^37^, ^38^ ^]^ Commonly used external phases, such as high‐molecular‐weight silicones or viscous oils, demand substantial energy to disperse stabilizing nanoparticles within the printed structures.^[^ ^39^, ^40^, ^41^, ^42^ ^]^ Consequently, external phases with tunable viscoelasticity and interfacial activity are desirable to accommodate diverse ink compositions while maintaining high resolution during patterning and allowing for a facile removal of the printed structures.

This study introduces a partially miscible system, consisting of an aqueous ink carrying nanomaterials, and the external 1‐butanol phase with suspended partially deacetylated chitin nanofibers modified with benzophenone (mChNF). In this system, mChNF serves dual roles: modulating interfacial assembly and tuning the rheological properties of the external phase. Specifically, electrostatic interactions between protonated mChNF in 1‐butanol and deprotonated graphene oxide (GO) in the aqueous ink generate a robust interfacial jammed layer. Furthermore, we demonstrate the versatility of this approach by combining the GO‐based aqueous ink with polymers and nanoparticles, resulting in an electrically conductive printed structure. Additionally, leveraging the partial miscibility of water and 1‐butanol and the ease of material incorporation, we develop hybrid inks combining GO and sodium alginate. During printing, these inks solidify as water exchanges with the external phase, facilitating the removal of printed 3D structures from the external phase.

## 2 Results and Discussion

Liquid‐in‐liquid printing involves shaping the stabilized liquid structure of functionalized materials into predefined 3D geometries within an external liquid phase. The fabrication of such an all‐liquid system requires: 1) interfacially active species in both the extruded liquid and the external phase that can complex at the interface to stabilize the tubular liquid structure in a non‐equilibrium form; 2) the external liquid bath must possess specific viscoelastic characteristics to hold the printed streams in place as they are extruded from the nozzle to enhance the printing resolution and shape fidelity; and 3) the printed structures must have sufficient mechanical strength to be removed from the external phase after printing.

Building on previous liquid‐in‐liquid printing studies,^[^ ^13^, ^43^, ^44^ ^]^ the study demonstrates the assembly of bio‐derived nanoparticles at the interface, enabling support‐free, high‐resolution printing. In this work, an aqueous suspension of graphene oxide is utilized as the primary ink (Figure S1, Supporting Information and discussion on the characterization of as‐synthesized GO), with GO exhibiting strong interfacial activity due to their hydrophobic basal planes and hydrophilic functional groups, such as hydroxyl, carboxyl, and epoxy groups.^[^ ^14^, ^15^, ^16^ ^]^

### 2.1 Interfacial Complexation and Jamming

**Figure 1:**
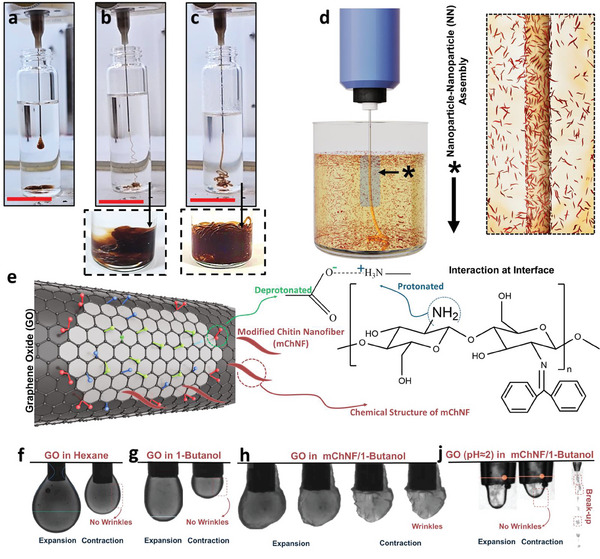
Photos showing the jetting of 1 mg mL^−1^ GO aqueous suspension into a) hexane, b) 1‐butanol, and c) 1‐butanol containing 1 mg mL^−1^ mChNF. Scale bare = 2.8 cm. The photos of liquid filaments after shaking are shown at the bottom of the images. Schematic illustrations of GO‐mChNF nanoparticle‐nanoparticle formation in the 1‐butanol external phase d) and the interaction of chitin nanofibers with GO at the interface e). Contraction of an aqueous GO pendant drop in f) hexane, g) 1‐butanol, h) 1‐butanol with 1 mg mL^−1^ mChNF, and j) an aqueous GO at pH≈2 (below the pKa of carboxylic acid) pendant drop in 1‐butanol with 1 mg mL^−1^ mChNF.

In conventional approaches, non‐polar liquids are typically employed as the external phase to limit GO diffusion from the aqueous stream. However, such systems require polymer‐based ligands, such as PSS‐[3‐(2‐aminoethyl) amino]propyl‐heptaisobutyl substituted POSS (POSS‐NH_2_), to stabilize the interface by inducing jamming.^[^ ^15^, ^32^ ^]^ In the absence of these ligands, the jetted GO stream succumbs to Plateau‐Rayleigh instabilities, fragmenting into droplets (**Figure** **1**a; Video S1, Supporting Information). To address this limitation, we propose using a partially miscible 1‐butanol phase as the external phase. This amphiphilic solvent temporarily stabilizes the system, as it contains both a non‐polar butyl chain and a polar hydroxyl group.^[^ ^45^, ^46^ ^]^ Liquid GO filaments can transiently form in 1‐butanol (Figure 1b; Figure S2, Supporting Information) and become pinned in the presence of partially deacetylated chitin nanofibers modified with benzophenone (mChNF) dispersed in 1‐butanol (Figure 1c–e; Video S1, Supporting Information). However, neat 1‐butanol is insufficient to maintain long‐term filament stability due to weak interfacial interactions, ultimately leading to filament breakup (Figure 1b). The synthesis and properties of mChNF are detailed in Figures S3 and S4 (Supporting Information). The deacetylation process activates amine groups that make a complex with GO, while benzophenone facilitates the dispersion of mChNF in 1‐butanol. Strong interfacial complexation between GO and mChNF, combined with interfacial jamming, results in a robust interfacial skin that stabilizes the extruded filament, even under intense agitation (Figure 1c–e; Figure S5, Supporting Information).

To evaluate interfacial complexation and jamming, a pendant drop of an aqueous GO suspension was immersed in three external phases: hexane, and 1‐butanol containing 1 mg mL^−1^ mChNF (Figure 1f–h). In hexane and pure 1‐butanol, no wrinkles were observed during drop contraction at the liquid‐liquid interface, indicating liquid‐like behavior without interfacial complexation (Figure 1f,g). However, in 1‐butanol with suspended mChNF, visible wrinkles formed on the droplet interface during contraction, signifying a solid‐like layer that buckles under stress (Figure 1h). The role of carboxylic acid in interfacial stabilization is further studied by adjusting the pH of the aqueous GO suspension below the pKa of carboxylic acid. As shown in Figure 1j, after lowering the pH of the aqueous GO suspension pH ≈2, no wrinkles were observed at the interface, and the liquid filaments failed to suppress Rayleigh instability. This behavior can be attributed to the lack of sufficient deprotonation at the interface. Despite the presence of other functional groups on the graphene oxide, they proved insufficient to stabilize the interface or promote liquid filament integrity. The permeability of the interface is visualized by tracking the diffusion of methylene blue from the GO suspension into the 1‐butanol phase (Video S2 and Figure S6, and discussion in Supporting Information). The observations confirm that mChNF enhances interfacial complexation and supports the formation of stable, high‐resolution structures.

### 2.2 Viscoelasticity of the External Phase

**Figure 2:**
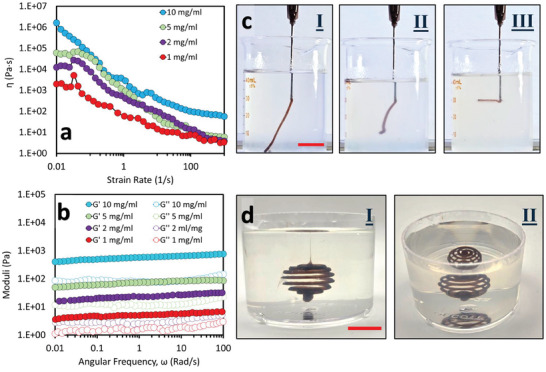
a) Flow curves showing the shear‐thinning behavior of mChNF/1‐butanol with varying mChNF concentrations (1–10 mg mL^−1^). b) Storage (*G*′) and loss (*G*″) moduli as a function of angular frequency for mChNF/1‐butanol at different concentrations. c) Photos of 10 mg mL^−1^ GO aqueous ink printing into mChNF/1‐butanol with mChNF concentrations of (I) 1, (II) 2, and (III) 5 mg mL^−1^, demonstrating that higher mChNF concentrations (5 mg mL^−1^) stabilize the printed structures, Scale bars = 1 cm. (d) GO ink printed into 5 mg mL^−1^ mChNF/1‐butanol, shown from a side view (I) and top view (II), demonstrating the role of external phase viscoelasticity in maintaining structural integrity in volumetric designs. Scale bars = 2 cm.

In addition to forming complexes with GO, mChNF imparts viscoelasticity to the 1‐butanol external phase, which is crucial for maintaining the shape fidelity of printed structures. To evaluate this viscoelasticity, rheological measurements were conducted at varying mChNF concentrations (**Figure** **2**a,b, droplet stabilization Video S3, droplet pinning Video S4, and streaming Video S5, Supporting Information). The flow curves of all mChNF/1‐butanol samples demonstrated shear‐thinning behavior, regardless of concentration (Figure 2a). This behavior, essential for liquid‐in‐liquid printing, arises from the disentanglement of mChNF nanofiber networks under shear.

Dynamic oscillatory measurements provided additional insights into the viscoelastic properties of the mChNF/1‐butanol external phase. Frequency sweep tests revealed the storage modulus (*G*′) and loss modulus (*G*″) as functions of frequency across different mChNF concentrations at 25 °C (Figure 2b). Both *G*′ and G″ increased with mChNF concentration but remained nearly frequency‐independent over the tested range. Notably, G′ consistently exceeded *G*″, indicating gel‐like behavior across all samples. This characteristic is fundamental for stabilizing printed structures during the liquid‐in‐liquid printing process.

The ability of mChNF/1‐butanol to support aqueous GO droplets was assessed immediately after droplet release (Figure S7 and Videos S3 and S4, Supporting Information) and again after 24 h (Figure S8, Supporting Information). As shown in Figure 2c, mChNF/1‐butanol containing 1 or 2 mg mL^−1^ of mChNF failed to stabilize the aqueous GO ink. However, as the mChNF concentration increased, interactions between nanofibers became more prominent. At concentrations exceeding 5 mg mL^−1^, the viscoelasticity of the external phase effectively stabilized the printed structures (see Figure 2c).

It is important to note that, in liquid‐in‐liquid printing, the rheological properties of the external phase play a more significant role compared to the ink itself. Unlike in direct‐ink printing, where the ink requires suitable shear‐thinning and viscoelastic properties, this approach primarily relies on the rheological behavior of the external phase for structural stability. In addition to the planar prints demonstrated so far (parallel to the XY‐plane), all liquid 3D model printing with overhanging features extending beyond the XY‐plane and incorporating movement across all 3D has also been achieved. Figure 2d and Figure S9 (Supporting Information) demonstrate that a Newtonian GO suspension can not only remain stable in a 5 mg mL^−1^ 1‐butanol/mChNF external phase but also maintain the integrity of spiral helix printed structures. Unlike the ink's rheological properties, which play a secondary role, the viscoelasticity of the external phase is critical for preventing the collapse or spreading of printed patterns.

Interestingly, as shown in Figure S10 and Video S6 (Supporting Information), highly viscous solutions lacking interfacial activity, such as GO in silicone oil, fail to stabilize printed patterns. This failure is attributed to Rayleigh‐Plateau instability, which leads to the breakup of liquid filaments. These findings highlight that both adequate interfacial complexation and the appropriate rheological properties of the external phase are essential to successfully print complex structures without the need for scaffolding or support.

### 2.3 Liquid‐in‐Liquid Printing Resolution

**Figure 3:**
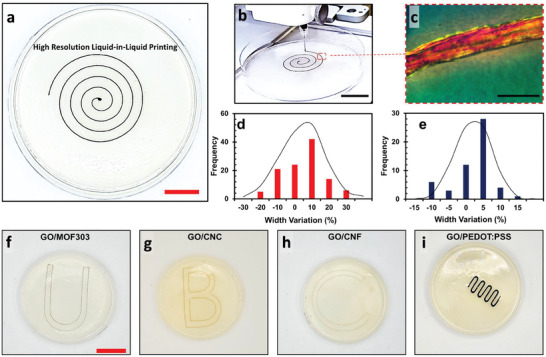
a,b) Liquid‐in‐liquid printing of a 10 mg mL^−1^ GO aqueous suspension using a 27‐gauge needle into a mChNF/1‐butanol external phase with 5 mg mL^−1^ mChNF. c) Polarized optical microscopy image of a liquid thread printed with a 27‐gauge needle. Width variation of printed samples using d) 27‐gauge and e) 16‐gauge needles, with GO and mChNF external phase concentrations of 10 and 5 mg mL^−1^, respectively. Structures printed with f) GO/MOF303, g) GO/CNC, h) GO/CNF, and i) GO/PEDOT, each with a 10 mg mL^−1^ ink concentration (50% GO, 50% listed material). Scale bars in (a–c) = 1.9, 1.6, and 0.5 cm; (f–i) = 3.4 cm.

To further investigate the printing resolution, a 10 mg mL^−1^ GO aqueous suspension was injected into 5 mg mL^−1^ mChNF/1‐butanol external phases using metal‐based needles with gauges 27 and 16, corresponding to inner diameters of 210 µm and 1194 µm, respectively (Video S7, Supporting Information). Figures 3a,b and Figures S11 and S12 (Supporting Information) demonstrate that successful printing can be achieved in both 5 and 10 mg mL^−1^ mChNF external phases. From the top view, the 27‐gauge needle provided excellent printing quality when using a 5 mg mL^−1^ mChNF/1‐butanol external phase (**Figure** **3**a). As 5 mg mL^−1^ mChNF is sufficient to stabilize the printed structures, this concentration was chosen to minimize material consumption. Figure S11 (Supporting Information) illustrates the printing of the same GO aqueous suspension in a 5 mg mL^−1^ mChNF/1‐butanol external phase using a 16‐gauge needle. The top‐view image of the printed structure confirms the same high‐quality resolution, demonstrating that the printing resolution is maintained regardless of needle size.

To quantitatively evaluate printing quality, samples printed with gauge 27 and 16 nozzles were analyzed using polarized optical microscopy, and the widths of the printed lines were recorded (Figure 3c; Figures S13 and S14, Supporting Information). For each nozzle size, measurements were taken at multiple locations, and a relative histogram of the line widths, normalized to the nozzle's inner diameter, was plotted to assess the variations. To further analyze the printing performance, a spreading parameter (S) was defined using the following equation:

(1) $$S=\frac{\mathrm{Width}\;\mathrm{of}\;\mathrm{printed}\;\mathrm{lines}-\mathrm{Inner}\;\mathrm{diameter}\;\mathrm{of}\;\mathrm{nozzle}}{\mathrm{Inner}\;\mathrm{diameter}\;\mathrm{of}\;\mathrm{nozzle}}\;\times 100$$

As shown in Figure 3d,e, the spreading parameters (S) for the samples printed with gauges 27 and 16 are ± 30% and ± 15%, respectively. These values are comparable to—and in some cases better than—the spreading parameters reported for 3D printing using viscoelastic inks through direct ink writing (≈30% to 120%).^[^ ^47^, ^48^, ^49^, ^50^, ^51^ ^]^ This demonstrates that GO‐mChNF offers a distinct advantage over direct ink writing, which typically relies on ink viscosity.^[^ ^52^, ^53^, ^54^, ^55^, ^56^ ^]^ Direct ink writing often faces challenges such as nozzle clogging, whereas the liquid‐in‐liquid printing method provides greater flexibility in processing without these issues.

Beyond achieving high printing resolution, the developed liquid‐in‐liquid printing method is highly versatile, enabling the creation of various structures using different nanomaterials. For example, Figure 3f–i show structures printed with GO combined with metal‐organic frameworks (MOF‐303), cellulose nanocrystals (CNC), cellulose nanofibers (CNF), and a conductive polymer (poly(3,4‐ethylenedioxythiophene) sulfonate, PEDOT:PSS). In all cases, the solid concentration of the aqueous ink was set to 10 mg mL^−1^, with half of the solid content composed of GO.

In this system, GO plays a crucial role in interfacial assembly, jamming, and stabilizing the liquid filaments. The secondary material, however, provides additional functionality to the printed structure. This approach becomes even more compelling when considering that materials such as CNF, CNC, and PEDOT:PSS, which lack the interfacial activity required to form liquid‐like filaments, can still be integrated through GO, as demonstrated in Figures S15–S18 (Supporting Information). For additional details on MOF‐303 characterization, refer to Figure S19 (Supporting Information). Consequently, GO serves as a universal carrier for other polymers and nanomaterials suspended in water in this versatile liquid‐in‐liquid printing process.

### 2.4 Liquid‐in‐Liquid Printing with Conductive Polymers

**Figure 4:**
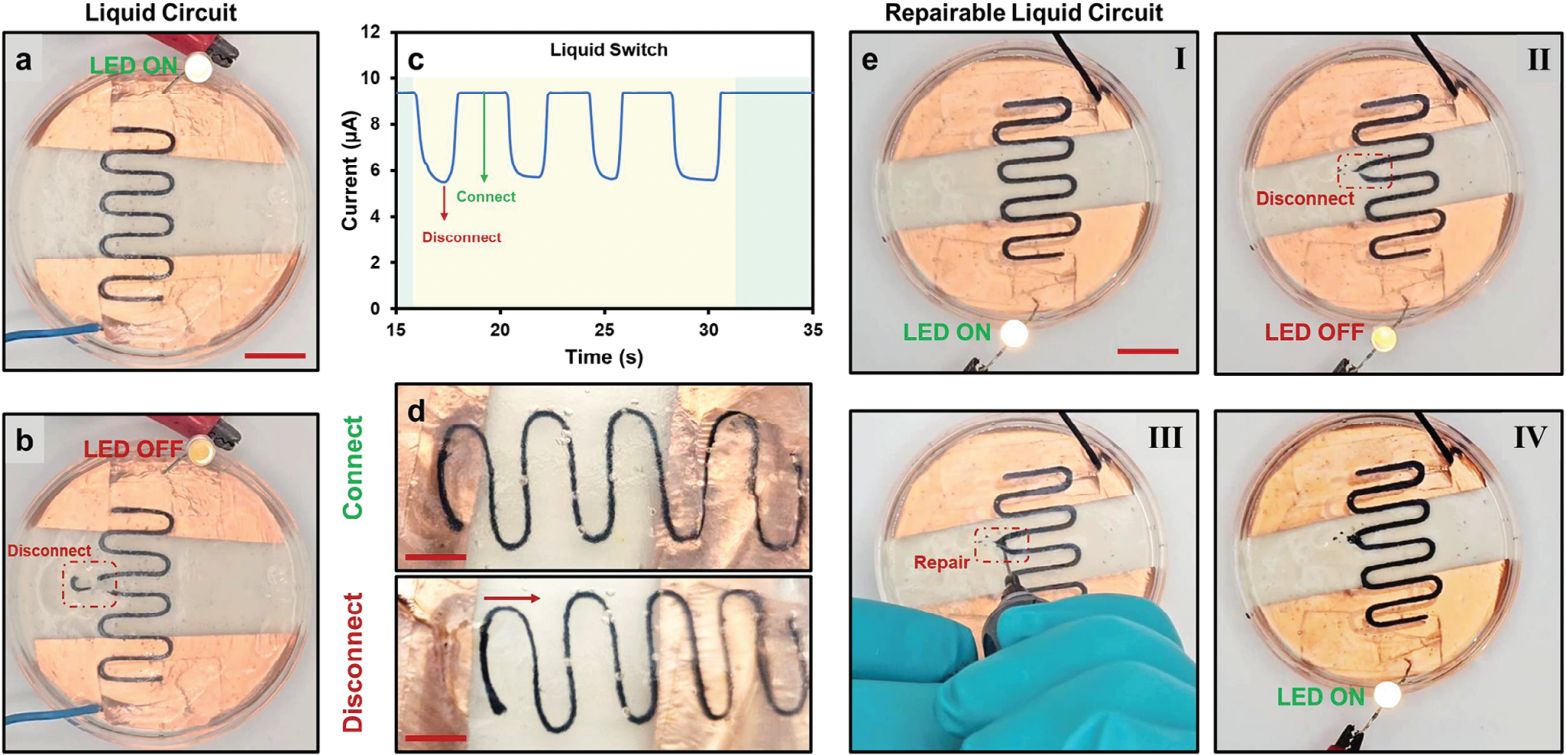
Demonstration of an all‐liquid electric switch using GO/PEDOT:PSS printed conductive pathways in mChNF external phase. a) The circuit in the ON state, where the LED is illuminated, indicates current flow through the conductive path. b) The circuit in the OFF state, where a disconnection in the printed path stops the current, turning off the LED. c) Sensor data showing the connection and disconnection process. d) Printed structure functioning as a switch through movement of the printed liquid. e) Repairability sequence: (I) circuit ON, (II) disconnection causing LED to turn OFF, (III) repair by injecting conductive ink, and (IV) circuit reactivated, demonstrating the switch's repeatability and repairability. Scale bars in (a), (a,b), (d), and (e) = 1, 0.5, and 1.2 cm, respectively.

The liquid‐in‐liquid printing method was used with conductive inks based on GO/PEDOT:PSS. An all‐liquid electric switch was demonstrated using GO/PEDOT:PSS conductive ink printed in the external phase (mChNF/1‐butanol, 5 mg mL^−1^). A conductive pathway that can be manipulated in real‐time, with the interfacial assembly and rheology of the external phase providing stability. This design showed that the conductive pathways remained operative while retaining flexibility (Video S8, Supporting Information). As shown in **Figure** **4**a,b, the print conductive paths successfully illuminated an LED. After establishing the circuit, the conductive structure was disconnected and reconnected, functioning effectively as a liquid‐based switch (Figures 4c, 6d; Video S8, Supporting Information). When the printed structure was disconnected, the current ceased, turning off the circuit. After disconnection in Figure 4c, the current did not drop to zero, as a small amount of water migrated through the interface and mixed with the external phase, increasing its ionic conductivity. Reinjecting the conductive ink at the liquid‐liquid interface restored the circuit (Figure 4e). The system's ability to recover functionality after disconnection without significant performance loss demonstrated its potential for reconfigurable electronics and recoverable circuits. The liquid conductive paths consistently maintained their electrical conductivity; they can recover from mechanical disruptions while preserving functionality, offering a promising solution for resilient electronic systems.

The liquid‐based electronic systems are promising alternatives to traditional solid‐state electronics, overcoming challenges related to flexibility, reconfigurability, and repairability.^[^ ^14^, ^57^ ^]^ These advancements are particularly relevant in wearable electronics, soft robotics, and sensing.^[^ ^58^, ^59^ ^]^ Here, we showed electronics that leverage conductive inks and liquid‐liquid interfaces to create circuits that were reconfigured and repaired, offering significant potential for future electronic systems.

### 2.5 Liquid‐in‐Liquid Printing with Alginate Coadjutant: Demolding and Core–Shell Morphologies

Although 10 mg mL^−1^ GO can be printed in the 1‐butanol/mChNF external phase with good resolution and shape fidelity, demolding the printed structures was challenging, which is essential for their use in solid forms. Hence, we incorporated a sodium alginate solution into the ink (sodium alginate to GO ratio of 50:50). The carboxylate groups in sodium alginate are expected to contribute to interfacial complexation with mChNF. However, when neat sodium alginate was used as the ink, the extruded material formed droplets instead of filaments (Figure S20, Supporting Information). In contrast, a stable filament was formed in the presence of GO, suggesting that GO effectively stabilizes the interface.

**Figure 5:**
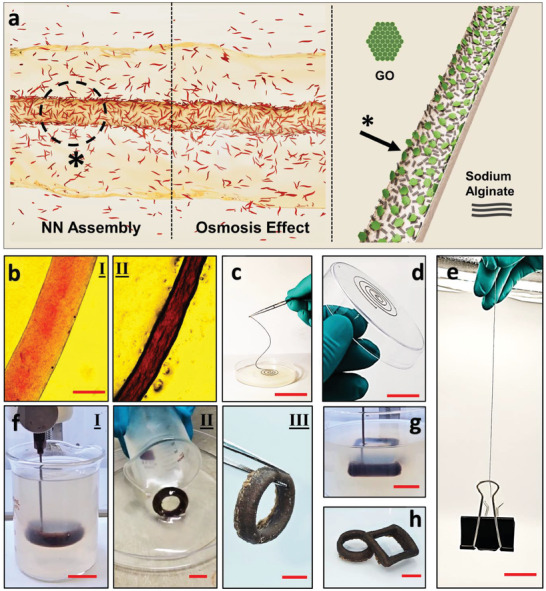
a) Schematic illustration of the interactions between GO/sodium alginate in an aqueous suspension that is extruded in mChNF/1‐butanol. b) Optical microscopy images showing water removal and solidification of GO/sodium alginate filaments at 1 and 15 min post‐printing (Scale bar = 0.5 cm). c) Solidified GO/sodium alginate filaments removed from the external phase (Scale bar = 5.1 cm), d) dried at RT while retaining shape (220 µm diameter, Scale bar = 3.5 cm), and e) demonstrating mechanical robustness by supporting a 25‐g load without structural failure (Scale bar = 2.3 cm). f) Volumetric liquid‐in‐liquid printing of cylindrical shape and demolding of printed structure. g) Volumetric printing of rectangular shape and h) de‐molded of solidified structure (Scale bars in (f–h) = 1 cm).

Highly water‐soluble sodium alginate is immiscible in 1‐butanol due to its strong hydration shell. During solvent exchange, water is gradually removed, causing alginate precipitation.^[^ ^60^ ^]^ This process, similar to wet spinning, is driven by osmotic effects at the semi‐permeable interface, facilitating controlled solidification.^[^ ^61^, ^62^ ^]^ As shown in Figure S21 and Video S9 (Supporting Information), a semi‐permeable interface supported water exchange with the 1‐butanol external phase via osmotic effects, facilitating the solidification of the structure (**Figure** **5**a,b). During solvent exchange, it solidifies as confirmed by polarized microscopy images in Figure 5b and Figure S22 (Supporting Information). As a result, the printed pattern can be easily removed from the external phase after just 15 min, retaining its shape due to the sufficient mechanical properties of the GO/mChNF network (Figure 5c; Video S10, Supporting Information). The filament dried while retaining its original shape (Figure 5d; Video S11, Supporting Information) and can withstand up to 25‐g loading (see Figure S23; Video S12, Supporting Information), revealing a strength of ≈175 MPa at ≈4% strain.

The spatial 3D printing process utilizes solvent exchange to solidify the printed structure without requiring any crosslinkers. As shown in Figure 5f and Video S13 (Supporting Information), the volumetric printed structure interacts with the external phase during the solvent exchange, facilitating its solidification. The process is versatile and not restricted by shape, as demonstrated by the robust cylindrical and rectangular pattern (Figure 5f–h). Both shapes were successfully de‐molded from the external phase without damage after solidification, highlighting the stability and mechanical integrity of the volumetric printed structures (Figure 5g,h).

**Figure 6:**
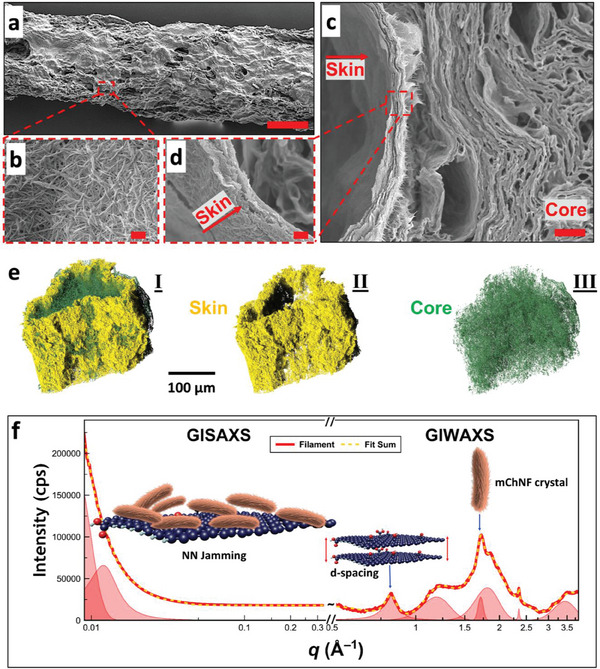
SEM images illustrating the morphology of GO/sodium alginate filament shells a–d), with mChNF pinned at the interface due to GO‐mChNF assembly and jamming. The filament core exhibits a distinct morphology from the shell. e) High‐resolution CT scans confirm a core–shell configuration, with a compact, dense shell surrounding a less dense core. f) GIWAXS and GISAXS data for the filament. Scale bars in (a), (b–d), and (c) = 100 µm, 300 nm, and 4 µm, respectively.

The morphology of the solidified filaments was analyzed to reveal the interfacial assembly and solidification involved in the formation of GO/sodium alginate printed structures. SEM images revealed a textured filament surface, which results from mChNFs being pinned at the interface through the GO/mChNF assembly and nanoparticle jamming. However, the filament's interior morphology differs significantly, exhibiting a core–shell configuration (**Figure** **6**a–d; Figures S24–S27, Supporting Information) that was confirmed by EDX analysis (Figures S28 and S29, Supporting Information).

High‐resolution CT scans further corroborated the core–shell structure, showing clear differences in density and compactness between the core and shell (Figure 6d; S25,S30, and S31, Supporting Information). The shell reveals a laminated morphology, distinguishable from the core. As reported in previous studies,^[^ ^57^, ^58^ ^]^ such laminated shell enhances mechanical stability and enables the removal of printed structures from the mChNF/1‐butanol external phase.

To confirm the interfacial complexation between GO and mChNF, XPS analysis provided compelling evidence in the hybrid filaments (detailed discussions and Figure S32, Supporting Information). Key findings included the presence of C‐N and *π–π* ^*^ interactions unique to the GO‐mChNF assembly, suggesting that mChNF locks the GO and stabilizes it at the filament surface. Additionally, nitrogen peaks corresponding to mChNF were detected at the filament interface, confirming that mChNF remains pinned at the surface due to interfacial interactions. Detailed XPS spectra (GO, mChNF, and the filament) and further analysis can be found in the SI.

Grazing‐incidence Small‐Angle X‐ray Scattering (GISAXS) and Grazing‐incidence Wide‐Angle X‐ray Scattering (GIWAXS) analyses were employed to investigate the structural features of GO, mChNF, and the hybrid GO‐mChNF filaments, revealing well‐defined larger‐scale spacing in GISAXS and finer crystalline ordering in GIWAXS. The unprocessed data and further discussions can be found in the Supporting Information, including Figures S33–S39 (Supporting Information).

To gain a deeper understanding of the interfacial interactions based on X‐ray Scattering, it is important to examine each nanomaterial separately. GISAXS of GO shows a peak at q = 0.036 Å⁻¹ (d‐spacing ≈19 nm), which confirms its 2D structure, while GIWAXS reveals defined interlayer spacing at 6.7 Å (Figure S39a, Supporting Information). In contrast, mChNF displays broader GISAXS peaks with a milder slope, reflecting its semi‐crystalline and fibrous nature (Figure S39b, Supporting Information). The GIWAXS data of mChNF shows a distinct peak at q = 1.36 Å⁻¹, corresponding to a d‐spacing of 4.6 Å, which is characteristic of the crystalline regions within mChNF, confirming its semi‐crystalline structure.

The GISAXS and GIWAXS patterns of the GO‐mChNF filaments confirm the successful assembly of the materials (Figure 6f). The patterns show structural features from both GO and mChNF, while also introducing new characteristics unique to the interfacial layer. The GISAXS data reveal a combination of larger‐scale spacing from both GO and mChNF, with peaks indicating interlayer distances ranging from 15.7 to 122.7 Å. However, there is a reduction in form factor oscillations, suggesting a more disordered assembly in the hybrid system. The GIWAXS data provide additional evidence of interfacial interactions, with new peaks appearing at q = 1.43 Å⁻¹ and q = 2.0 Å⁻¹. These peaks indicate the formation of denser structural features due to electrostatic complexation between GO and mChNF, leading to a rearranged complex structure. The shifts in peak positions and intensities further suggest the creation of a denser layer at the interface.

X‐ray studies also offer insights into the orientational alignment of mChNF at the interface. When mChNF assembles rapidly, it forms a more random orientation, which helps suppress Rayleigh‐Plateau instabilities. In contrast, a slower stabilization process results in a more aligned structure with a higher orientation index. As shown in Figure S40 (Supporting Information), azimuthal intensity profiles reveal these alignment differences. The cross‐section of the mChNF film (which dries slowly) shows high alignment (R ≈ 0.75), while the GO‐mChNF filament at the surface exhibits an isotropic structure with a less defined profile (R ≈ 0.15). This difference is due to the rapid interfacial assembly of mChNF onto the GO surfaces (the calculation method based on the Hermans order parameter is available in the Supporting Information)

## 3 Conclusion

We introduced a method for high‐resolution liquid‐in‐liquid printing at water/1‐butanol interfaces through the electrostatic complexation of graphene oxide (GO) in the extruded ink and modified chitin nanofibers (mChNF) in the external phase. The incorporation of mChNF enhances the stability of interfacial complexation and shape integrity. By controlling the rheological properties of the mChNF/1‐butanol phase, the approach enables support‐free, precise, and stable printing of filaments. The high‐resolution printing achieved in this system results from the combined effects of interfacial complexation and the rheology of the external phase. These factors contribute to the formation of a durable interfacial “skin” that stabilizes printed shapes and prevents typical instabilities observed in liquid systems, preserving shape fidelity.

Characterization of the GO‐mChNF interface, using XPS, Micro‐CT, GISAXS, and GIWAXS, confirmed the successful formation of a jammed interfacial network with a distinct core–shell morphology, reinforcing both structural and mechanical stability. This method is also highly adaptable, allowing for the incorporation of hybrid inks that combine GO with materials like sodium alginate, metal‐organic frameworks, cellulose nanoparticles, and conductive polymers, thereby extending the functionality of the printed materials. For instance, utilizing GO: sodium alginate inks enables the demolding of volumetric structures through support‐free printing via solvent exchange, highlighting its versatility and practical applications. This versatility enables the production of soft materials with tunable mechanical and conductive properties, opening up new possibilities for applications in flexible electronics and reconfigurable sensors.

In summary, this work provides a comprehensive framework for the liquid‐in‐liquid printing approach by utilizing the interfacial complexation of mChNF/GO assemblies, enabling high‐resolution volumetric fabrication without external scaffolds or surfactant‐based stabilization. The integration of osmosis‐driven solvent exchange introduces a new demolding strategy. By combining nanoparticle interfacial assembly with external‐phase rheology control, this structured liquid system offers new possibilities for wearable electronics, soft robotics, and additive manufacturing.

## 4 Experimental Section

The Supporting Information includes a detailed experimental section covering the materials and instruments used, methods for synthesizing modified, GO, mChNF, CNF, and MOF‐303, as well as the fabrication of structured liquids and hybrid inks. It also features descriptions of tests such as expansion/contraction and X‐ray analysis. The characterization section provides information about GO, mChNF, and MOF, supported by XPS, GISAXS, and GIWAXS data and discussions. Additionally, the document contains extensive supporting figures to complement the main text, presenting the methodologies and findings more clearly.

## Conflict of Interest

The authors declare no conflict of interest.

## Supporting information



Supporting Information

Supplemental Video 1

Supplemental Video 2

Supplemental Video 3

Supplemental Video 4

Supplemental Video 5

Supplemental Video 6

Supplemental Video 7

Supplemental Video 8

Supplemental Video 9

Supplemental Video 10

Supplemental Video 11

Supplemental Video 12

Supplemental Video 13

## Data Availability

The data that support the findings of this study are available in the supplementary material of this article.
