# Supplementary material for: Liquid Printing in Nanochitin Suspensions: Interfacial Nanoparticle Assembly Toward Volumetric Elements, Organic Electronics and Core–Shell Filaments
Source: Small Methods. 2025 Mar 3;9(8):2500100. doi: 10.1002/smtd.202500100 (PMC12391653; doi:10.1002/smtd.202500100)
Supplement: Supplementary file 1 — Supporting Information [file SMTD-9-2500100-s007.pdf]

small methods

Supporting Information

for *Small Methods*, DOI 10.1002/smtd.202500100

Liquid Printing in Nanochitin Suspensions: Interfacial Nanoparticle Assembly Toward Volumetric Elements, Organic Electronics and Core–Shell Filaments

Mahyar Panahi-Sarmad, Ahmadreza Ghaffarkhah, Lukas Alexander Bauman, Amin Babaei-Ghazvini, Seyyed Alireza Hashemi, Bishnu Acharya, Boxin Zhao, Mohammad Arjmand, Feng Jiang* and Orlando J Rojas**

Supporting information:

Liquid printing in nanochitin suspensions: Interfacial nanoparticle assembly toward volumetric elements, organic electronics and core-shell filaments

Mahyar Panahi-Sarmad [a,b,#], Ahmadreza Ghaffarkhah [b,c,d,#,*], Lukas Bauman [b,e,#], Amin Babaei-Ghazvini [f], Seyyed Alireza Hashemi [d], Bishnu Acharya [f], Boxin Zhao [e], Mohammad Arjmand [d], Feng Jiang [a,b,*], Orlando J. Rojas [a,b,c,g,*]

[a] *Department of Wood Science, The University of British Columbia, 2424 Main Mall #2900, Vancouver, BC V6T 1Z1, Canada*

[b] *Bioproducts Institute, University of British Columbia, 2385 East Mall, Vancouver, BC V6T 1Z4, Canada.*

[c] *Department of Chemical and Biological Engineering, University of British Columbia, 2360 East Mall, Vancouver, BC V6T 1Z3, Canada.*

[d] *Nanomaterials and Polymer Nanocomposites Laboratory, School of Engineering, University of British Columbia, Kelowna, BC, V1V 1V7, Canada*

[e] *Department of Chemical Engineering, University of Waterloo, 200 University Avenue West, Waterloo, Ontario N2L 3G1, Canada*

[f] *Department of Chemical and Biological Engineering, University of Saskatchewan, 57 Campus Drive, Saskatoon, Saskatchewan, S7N5A9, Canada*

[g] *Department of Chemistry, The University of British Columbia, 2036 Main Mall, Vancouver, BC V6T 1Z1, Canada*

*Corresponding authors: A.G.: ah.ghaffarkhah@ubc.ca; F.J.: feng.jiang@ubc.ca; O.J.R: orlando.rojas@ubc.ca

[#]These authors have contributed equally to this work.

Contents

1. Experimental Section 3
1.1. Materials and Instruments 3
1.2. Methods 4
1.2.1. Synthesis of Graphene Oxide (GO) 4
1.2.2. Synthesis of modified Chitin Nanofibers (mChNF) 4
1.2.3. Synthesis of Cellulose Nanofibers (CNF) 6
1.2.4. Synthesis of MOF-303 6
1.2.5. Fabrication of Structured Liquids 7
1.2.6. Hybrid Inks 8
1.2.7. Expansion/Contraction Test 8
1.2.8. Methods of X-ray Analysis 9
2. Characterization and Discussion 11
2.1. Characterization of GO 11
2.2. Characterization of mChNF 12
2.3. Characterization of MOF 14
2.4. XPS Analysis and Discussion 15
2.5. X-ray scattering and Discussion 16
2.5.1 GISAXS and BIFT Analysis Discussion 16
2.5.2. GISAXS + GIWAXS Discussion 18
3. Supporting Figures 21
4. References 54

1. Experimental Section

1.1. Materials and Instruments

In this study, graphite flakes' exfoliation and subsequent graphene oxide (GO) synthesis are carried out using large flake graphite supplied by Asbury Carbons and analytical grade reagents. These reagents include H_2SO_4 (highly concentrated sulfuric acid, 98 %), H3PO4 (orthophosphoric acid, 85%), H_2O_2 (hydrogen peroxide, 37%), HCl (hydrochloric acid, 37%), ethanol, and KMnO4 (potassium permanganate), supplied by Sigma-Aldrich. Ultra-pure deionized water was used in all of the stages of the synthesis, generated via ELGA, MEDICA EDI 15/30, with a resistivity of 18.2 MΩ. For the production of chitin nanofibers (ChNF), raw shrimp chitin was procured, and purification steps were carried out using HCl, NaOH, and H_2O_2. Further reagents like acetic acid and benzophenone were used to modify ChNF chemically. For the synthesis of MOF-303, the reagents used were 3,5-Pyrazoledicarboxylic acid obtained from Sigma-Aldrich, Aluminum Chloride Hexahydrate, and Sodium Hydroxide (NaOH) sourced from Fisher Scientific. These materials are essential for facilitating the coordination of metal ions and the organic linker, forming the MOF structure under controlled conditions. TEMPO (2,2,6,6-tetramethylpiperidine-1-oxyl radical, 99.9%), sodium hypochlorite (NaClO, 11.9%), and sodium bromide (NaBr, >99%) were obtained from Sigma-Aldrich and used without further purification. PEDOT:PSS (Clevios, PH1000) aqueous dispersion (1–1.3 wt%) was purchased from Heraeus. Cellulose nanofibrils (CNF) were synthesized through TEMPO-mediated oxidation, utilizing 10 mmol of NaClO per gram of cellulose derived from northern bleached softwood kraft pulp (NBSK, Canfor Corporation). Cellulose Nanocrystals (CNC) were purchased from CelluForce and used without further purification. Tensile testing was performed using the Instron Universal Mechanical Testing System, and theological properties were measured using the Anton Paar MCR 302 Rheometer.

GIWAXS measurements were performed at the Brockhouse X-ray Diffraction Sector of the Canadian Light Source (CLS), specifically using the low-energy wiggler (BXDS-WLE) beamline with a photon energy of 15.1 keV (λ = 0.81931 Å).

1.2. Methods

1.2.1. Synthesis of Graphene Oxide (GO)

The synthesis of well-exfoliated GO is carried out following the improved Hummers method reported by Marcano et al. [1], with some slight modifications because of the nature of the used large flake graphite source according to our previous studies [2–4]. Accordingly, the acidic medium is prepared upon mixing H_2SO_4 and H_3PO_4 with a ratio of 9:1 vol:vol (360 mL:40 mL), followed by the addition of 3 g graphite and subsequent mixing for 30 min. Next, the temperature of the mixture declined to about ~5 °C using an ice bath, and 18 g $KMnO_4$ was slightly added to the cooled mixture while stirring. The temperature was then elevated to 55 °C using an oil bath and maintained at this temperature for 18 h while stirring to fully oxidize the graphite flakes. The resulting brownish acidic mixture was cooled down to room temperature, followed by the addition of 800 mL:5 mL H_2O:H_2O_2 and subsequent vigorous stirring. The exfoliated GO flakes were stirred for a further 2 h and applied to purification steps. These steps include sequential washing with water, 30 vol% HCl, ethanol, and water to remove the impurities. The purified mixture was thence diluted with deionized water and bath sonicated for 1 h. The obtained GO suspension was concentrated through centrifugation at 11000 rpm for 1 h and stored till further use.

1.2.2. Synthesis of modified Chitin Nanofibers (mChNF)

1.2.2.1. Chitin Purification

In a typical procedure (see Figure S1), 300 g of raw shrimp chitin was subjected to demineralization by stirring in 4 L of 1M HCl for 12 hours. The chitin was then filtered and washed

thoroughly with ultrapure water until the pH of the filtrate reached neutral. Following demineralization, the sample underwent deproteination by stirring in 4 L of 1M NaOH for 12 hours, after which the pH was again brought to neutral through extensive rinsing with ultrapure water. To further purify the chitin, the sample was bleached in 3 L of 5% H_2O_2 at 90°C for 3 hours under continuous stirring. Afterward, the bleached chitin was filtered and rinsed with ultrapure water to remove any remaining bleaching agents.

1.2.2.2. ChNF Production

After purification, the chitin was deacetylated by stirring in 2 L of 20 wt% NaOH at 90°C for 3.5 hours. The deacetylated chitin was then filtered and washed with ultrapure water until the pH reached neutral. The resulting product was dispersed in a 1% acetic acid solution (1% w/v) to a dry weight of 1% and mixed using a household blender to create a fine dispersion. This dispersion was passed through a microfluidizer at a pressure of 32,000 psi for two passes using a z-style reaction chamber. The resulting chitin nanofiber suspension was then freeze-dried for storage and further use.

1.2.2.3. Benzophenone Modification

For chemical modification, 50 g of the freeze-dried chitin nanofibers were redispersed by homogenization at 15,000 rpm in a solution of 90:10 ethanol acid (v/v), along with the addition of 100 g of benzophenone. The solvent was subsequently removed via rotary evaporation, and the resulting composite was cured at 105°C overnight to promote the formation of a Schiff base. Following the Schiff base formation, the composite was redispersed in 300 mL of acetic acid and stirred at 50°C for 3 hours to dehydrate and form imine bonds. The modified chitin was purified through three cycles of centrifugation at 10,000 RCF, ensuring the supernatant did not precipitate when added to water. Finally, the purified concentrate was redispersed in butanol at a concentration

of 10 mg/mL and passed through the microfluidizer again at 32,000 psi to produce the final modified chitin nanofiber dispersion.

1.2.3. Synthesis of Cellulose Nanofibers (CNF)

CNF was synthesized from Northern Bleached Softwood Kraft (NBSK) pulp through TEMPO-mediated oxidation. In this process, sodium hypochlorite (NaClO) was added at a rate of 10 mmol per gram of pulp, along with catalytic amounts of TEMPO (2,2,6,6-Tetramethylpiperidine-1-oxyl) and sodium bromide (NaBr). The mixture was then subjected to high-speed blending using a household blender (TNC5200, Vitamix, USA) for 25 minutes to achieve uniform oxidation and fibrillation of the cellulose [5]. Following the blending, the CNF dispersion was concentrated by gentle heating on a hot plate with continuous stirring to remove excess water. The concentrated CNF was then filtered through a nylon cloth with a mesh size of 300 to remove larger particulates. The final concentration of the CNF dispersions was adjusted based on the specific experimental requirements.

1.2.4. Synthesis of MOF-303

MOF-303 was synthesized using a solvothermal method with a slight modification for optimization. In a typical procedure, 1-H-pyrazole-3,5-dicarboxylic acid (PZDC) and NaOH were dissolved in deionized water, followed by the slow addition of aluminum chloride hexahydrate ($AlCl_3 \cdot 6H_2O$). The solution was stirred continuously until a milky white precipitate formed, indicating the initial formation of MOF-303. The reaction mixture was then transferred to a sealed reaction vessel and heated at 120°C for 24 hours. After cooling to room temperature, the solid product was collected via centrifugation and washed several times with deionized water and ethanol to remove any unreacted species [6].

1.2.5. Fabrication of Structured Liquids

GO aqueous suspensions were prepared and injected into a 1-butanol bath containing mChNF. Using a BioX 3D printer, structured liquid filaments were fabricated via interfacial complexation. Various concentrations of GO and mChNF/1-butanol were tested to explore the impact of nanoparticle interactions at the interface.

1.2.5.1. GO Streams into mChNF external phase

GO aqueous suspensions at concentrations of 1 mg/mL and 10 mg/mL were loaded into a plastic syringe and extruded into a 1-butanol bath containing mChNF at concentrations of 1, 2, 5, and 10 mg/mL. This was performed using a BioX 3D printer. The GO aqueous suspension, when injected into the 1-butanol, initially formed liquid streams, although they were inherently unstable. For comparison, this structure could not be generated in hexane due to the absence of interfacial assembly, resulting only in the formation of discrete droplets. The same droplet behavior was observed when aqueous CNF and CNC (cellulose nanocrystal) suspensions were injected due to weak electrostatic interactions at the interface. The morphology and interlocking properties of CNF and CNC with mChNF play a critical role in determining the stability and structure formation.

By utilizing GO concentrations between 5-10 mg/mL and mChNF concentrations of 1, 2, 5, and 10 mg/mL, stable GO-liquid threads were achieved. This stability was due to the nanoparticle-interlock-nanoparticle jamming assembly between the GO and mChNF at the interface, which created an elastic interfacial skin around the extruded threads. This skin prevented the aqueous jet from breaking into droplets, thereby suppressing the Plateau-Rayleigh instability that would otherwise destabilize the stream. The specific needle inner diameter (ID) and extrusion pressure parameters need to be optimized for each system.

1.2.6. Hybrid Inks

To further explore the potential of GO in conjunction with other nanomaterials (1:1 ratio), hybrid aqueous inks were developed by combining GO with other functional nanoparticles such as CNF, CNC, metal-organic frameworks (MOF-303), and PEDOT:PSS. These hybrid inks exploit the complementary properties of GO and the other materials, providing enhanced functionality for advanced printing applications.

1.2.6.1. Structured Liquids with Hybrid Inks

Hybrid inks were printed using a 3D Bioprinter, allowing precise control over the printing parameters to produce structures with tailored properties. A gauge 27 dispensing needle was employed for extrusion, and the pressure was carefully controlled within a range of 10-30 kPa, depending on the specific material and application. The flexibility of this process also permits manual extrusion using a simple compressor, enabling customized designs for various applications. The method allows for the fabrication of hybrid structures that leverage the individual properties of the combined materials to achieve unique functionalities in printed devices.

1.2.7. Expansion/Contraction Test

An expansion/contraction test was conducted using a pendant drop tensiometer to evaluate the interfacial complexation and jamming behavior of GO-mChNF at the water/1-butanol interface. In this experiment, an aqueous suspension of GO (1 mg/mL) was tested in three different external phases to observe interfacial behavior under various conditions: The concentration of both mChNF and GO was set at 1 mg/mL to ensure consistency in light transmission for pendant drop analysis. Wrinkling and stability observations were recorded for each condition, demonstrating the influence of mChNF on interface stabilization. While this setup illustrated the solid-like behavior

at the water/1-butanol interface with mChNF-GO, it was unsuitable for reliable interfacial tension (IFT) measurements due to continuous water flux from the GO droplet into the 1-butanol bath, resulting in constant droplet curvature changes. Therefore, IFT data were not recorded.

1.2.8. Methods of X-ray Analysis

The 2D scattering data, captured at an incidence angle of 0.5º, were recorded by a Rayonix MX300 CCD detector (pixel size: 73.242 μm × 73.242 μm), positioned 344.57 mm from the sample. Calibration was done using a Lanthanum boride (LaB6) standard, and data analysis and visualization were conducted using the GSAS-II software [7]. Grazing-incidence small-angle X-ray Scattering (GISAXS) experiments were performed at the CLS on the BXDS-WLE beamline. The photon energy was set at 15.1 keV using a Si(111) monochromator. Beam dimensions were controlled by slits with vertical and horizontal gaps of 0.2 mm and 0.3 mm, respectively, and the incident angle was accurately adjusted to 0.1°. The GISAXS patterns were recorded with a Rayonix MX300 CCD detector (pixel size: 73.242 μm) positioned 2506 mm away from the sample. Calibration was done using a silver behenate standard, and data analysis was performed using the GSAS-II software [7], incorporating both polarization and solid-angle corrections. Parts of SAXS data, including GISAS-II processed data, were post-processed using the BioXTAS Raw software. Guinier analysis was applied to determine the radius of gyration (R_g), maximum particle dimension D_{max}, and the pair distribution function P(r) [8,9]. The Hermans order parameter, denoted as S (where $0 \leq S \leq 1$), is used to quantify the degree of order within a system, calculated according to Equations1-3. A value of S = 0 corresponds to an isotropic system (random organization), while S = 1 represents a perfectly aligned anisotropic structure [10]. For this study, 2D patterns of the films were recorded by taking measurements at five different points across each colored region. The order parameter was then determined using this approach, as outlined in previous research [11].

Data analysis and visualization were carried out using the GSAS-II software package [7]. XPS spectra of the prepared samples were collected using an AXIS Supra instrument with a 500 mm Rowland circle monochromated Al Kα X-ray source and postprocessing with Casa XPS software [12].

$$S = \frac{3\langle \cos^2 \gamma \rangle - 1}{2} \quad (1)$$

$$\langle \cos^2 \gamma \rangle = 1 - 2\langle \cos^2 \varphi \rangle \quad (2)$$

$$\langle \cos^2 \varphi \rangle = \frac{\int \mathrm{I}(\varphi) \cos^2 \varphi \sin \varphi \, \mathrm{d}\varphi}{\int \mathrm{I}(\varphi) \sin \varphi \, \mathrm{d}\varphi} \quad (3)$$

CT scan sample preparation: No extensive preparation was required for this imaging technique, where samples such as filament segments with a thickness of 0.5 mm were scanned non-invasively. Four filament samples were randomly selected and placed inside a capillary tube. The ends of the capillary tube were sealed with reusable adhesive to prevent any moisture loss during scanning, and the tube was then placed on the sample stage. The sample was carefully aligned on the stage to ensure it remained within the beam's field of view. A rotating stage was used to adjust the sample orientation in 0.06° increments relative to the incident white beam at 20 keV. To achieve better contrast through edge enhancement in phase contrast imaging, the distance between the sample and detector was maintained at 5 cm. Detailed principles and theory of X-ray phase contrast imaging can be found in previous studies [13,14].

The GISAXS data were analyzed using the RAW software package, which provides a robust platform for processing and analyzing small-angle scattering data. The software allows for the extraction of key parameters such as the radius of gyration R_g, the maximum particle dimension D_{max}, and the pair distribution function P(r) using the Bayesian Indirect Fourier Transformation (BIFT) method. The analysis begins with the initial data correction, including background subtraction and scaling, followed by the Guinier approximation for small q-values, which is used to estimate R_g. The BIFT method was then applied to extract P(r), which provides insights into the real-space distribution of scattering entities. The goodness-of-fit was evaluated using chi-squared statistics, ensuring that the model sufficiently described the experimental data. RAW software also facilitated residual analysis, verifying the quality of the fits across the q-range. This process ensures that the derived structural information is reliable and consistent with the experimental observations [15].

2. Characterization and Discussion

2.1. Characterization of GO

The exfoliated GO flakes were analyzed using diverse techniques to confirm their successful formation with expected functionalities and features for interfacial complexation. The outcome of X-ray diffraction (Figure S1a)) showcased a sharp peak at 2Θ of 10.53°, corresponding to the interlayer spacing (d-spacing) of ~8.4 Å [16,17]. This peak corresponds to the (001) crystalline plane of GO, confirming an increase in the interlayer spacing compared with graphite flakes (3.35 Å), revealing the generation of oxygen-based moieties on the surface of GO. This result was further confirmed by the FTIR analysis (Figure S1 b), showing the existence of oxygen-based functionalities on GO, including hydroxyl (-OH) (3382 cm^{-1}), carbonyl (C=O) (1735 cm^{-1}), double

bonds carbon-carbon (C=C) (1623 cm^{-1}), C-OH (1365 cm^{-1}), C-O-C (1222 cm^{-1}), and C-O (1060 cm^{-1}) [18,19]. These functionalities, especially the carboxylic acid functional groups of GO, are essential to obtain a uniform dispersion of GO in water and facilitate the nanoparticles jamming at the interface upon interaction with positively charged ligands.

The micro-Raman spectroscopy results also confirmed the successful formation of GO flakes with minimized defects (Figure S1 c). This analysis is a potential tool for structural or chemical defects analysis owing to its phonon modes. In this case, the two main fingerprint peaks of GO, namely D-band and G-band, were observed at 1347 cm^{-1} and 1589 cm^{-1}, respectively. The D-band corresponds to the graphene flakes disorders, activated through the single-phonon interval scattering process. Meanwhile, the G-band, in accordance with the C=C peak of FTIR, reveals the vibration of sp*2*-bonded carbon atoms in the graphene lattice, showing the graphitic nature of these flakes [20]. The ID/IG ratio of the synthesized GO flakes, as a numerical insight into the GO defects, was measured to be ~0.86, indicating the controlled rate of defects during the synthesis. The HRTEM analysis further validated the quality of the synthesis, showing the formation of large 2D GO flakes with minimized defective sites (Figure S1 d-e). These outcomes confirmed the successful exfoliation of GO with the required features toward interfacial complexation.

2.2. Characterization of mChNF

To confirm the benzophenone modification had not significantly affected the structure of the chitin nanofibrils, the modified chitin was compared to unmodified chitin with the same number of microfluidizer passes to remove factors the mechanical treatment had on the structural and chemical properties (Figure S4 a) Atomic force microscopy was performed to elucidate any structural differences between the 2 materials. While the dispersion of the samples on mica is noticeably different for unmodified (seen Fig S4 b-c) and modified (Fig S4 d-e), this could be

attributed to differences in the dispersion solvents of water vs. 1-butanol for unmodified and modified, respectively. When the individual nanofibers are observed, the benzophenone modification did not significantly change the structure of the chitin, with both samples showing a dispersion of lengths between 250-1000 nm and diameters in the range of 30 nm. While the structure was not significantly affected by the modification, the optical and chemical properties were. UV-Vis was performed in support to confirm the presence of benzophenone on the chitin nanofibers. Fig S4 g to look for the characteristic absorption of benzophenone. Unlike the unmodified chitin with little absorption 240-300 nm range, the modified chitin demonstrates an additional peak due to the presence of benzophenone. It should be noted that this method cannot determine the degree of modification, as the C=O bond responsible for benzophenone's absorption spectra is converted to a C=N, preventing quantification by this method. FTIR was also performed on the modified and unmodified chitin with no significant differences in the absorption spectra, which was attributed to the low modification density and the C=O peaks from acetyl groups at 1690 nm-1 obscuring the C=N peaks from 1640-1690 nm-1. Conductometric titration was used to protonate the primary amine groups to find the degree of deacetylation and degree of modification. After modification, the primary amine groups are converted to imines and do not protonate at the same pH as primary amines. By fitting the curves of the conductometric titrations, it was found that for 10mg of unmodified chitin, 0.304 ml of 0.02 M NaOH was needed to deprotonate the amine groups. In contrast, 0.175 ml was required for the modified chitin. Using the following equation, $DDA=203*[((\Delta V)*0.02)/((M_{chitin})+42*(\Delta V*CN_{aOH}))]*100$ where ΔV refers to the difference in volume between the descending and ascending slopes of the conductivity, M_{chitin} refers to the mass of chitin tested, C_{NaOH} refers to the molarity of the NaOH solution we can calculate the degree of deacetylation. The calculated degree of deacetylation was 12% for the

unmodified chitin, while the modified measured 6.5%. Due to the relatively mild conditions used for deacetylation, a degree of deacetylation of 12% was within the range of expected values, albeit relatively low for deacetylated chitin nanofibers. Furthermore, these results enabled quantification of the degree of modification. Since the measured DDA% decreased by 46%, we can confirm that the benzophenone forms a Schiff base with the deacetylated amine groups and reduces their quantity. Finally, while 46% modification is not exceptional, a high degree of modification in this application is undesirable. This is due to the interfacial activity that primary amines provide to chitin nanofibers. If all the primary amine groups were modified, the interfacial activity of these fibers would suffer.

2.3. Characterization of MOF

The crystallinity of MOF-303 was confirmed using powder X-ray diffraction (PXRD). The PXRD patterns obtained were consistent with the simulated pattern for MOF-303, indicating a high level of crystallinity across all synthesis methods. The characteristic peaks corresponding to the [Al(OH)(PZDC)] structure were observed, confirming the successful formation of the MOF. This crystallinity was retained even when synthesized on different scales (solvothermal, reflux, vessel, and microwave methods), as evident from the overlay of experimental and simulated diffraction patterns, showing no significant deviations in peak positions or intensities.

Nitrogen adsorption isotherms at 77 K were measured to determine the specific surface area and porosity of the MOF-303 samples. The BET surface areas of the MOF-303 samples were found to exceed 1300 m^2/g across the different synthesis methods. Specifically, the solvothermal method yielded a surface area of 1342 m^2/g, while the reflux and vessel methods provided values of 1384 m^2/g and 1380 m^2/g, respectively. The microwave method resulted in a slightly lower surface area

of 1307 m^2/g. These results demonstrate the MOF's high porosity, making it well-suited for various adsorption applications.

2.4. XPS Analysis and Discussion

The X-ray Photoelectron Spectroscopy (XPS) analysis of GO, mChNF, and the hybrid GO-mChNF filaments provides compelling evidence of the interfacial assembly between these nanomaterials. The C1s spectra of GO (Figure S32a) reveal distinct peaks corresponding to C=O at ~288.5 eV, C-OH at ~286.4 eV, C-O-C at ~285.7 eV, and the π-π* interaction at ~291 eV, consistent with the oxidation and functionalization of GO with various oxygen-containing groups. The O1s spectra further show contributions from different oxygen-containing species, indicating the presence of hydroxyl and epoxy groups, contributing to the amphiphilic behavior of GO. For mChNF (Figure S32 b), the deconvoluted C1s spectra show the characteristic peaks, including contributions from C-O and C-N bonds at ~286.6 eV and C=O or N-acetyl groups at ~288.0 eV, confirming the preservation of chitin's structural integrity during the modification process. The O1s spectra of mChNF exhibit similar binding energy positions to GO but with a relatively higher proportion of amine-containing groups due to the deacetylation of chitin nanofibers, which are essential for promoting interfacial complexation with GO.

The XPS spectra of the GO-mChNF hybrid filaments (Figure S32c) provide further confirmation of this interfacial complexation. The C1s spectra exhibit multiple peaks attributed to C-H, C-C, and C-N bonds at ~284.6 eV and ~285.4 eV, consistent with contributions from both mChNF and GO. Most importantly, the persistence of the π-π* interaction at ~291 eV in the hybrid filaments further supports the localization of GO at the interface. This interaction is critical, as it confirms the alignment of GO at the interface, driven by the conjugated π systems, reinforcing the filament structure through interfacial stabilization. Moreover, the observed N1s peak from the amine groups

of mChNF, absent in pure GO, provides direct evidence of electrostatic interactions between the deprotonated carboxyl groups of GO and the protonated amine groups of mChNF. These interactions lead to the jamming of GO and mChNF at the interface, stabilizing the liquid-liquid printed filaments and ensuring the formation of a robust interfacial "skin" that can withstand external forces.

2.5. X-ray scattering and Discussion

2.5.1 GISAXS and BIFT Analysis Discussion

In this study, we performed GISAXS to investigate the nanostructural properties of three different samples: Filament, GO, and Chitin. The analysis was supported by the BIFT method to extract the pair distribution function P(r) and further refine the real-space characteristics of the scattering particles. The pair distribution function P(r) provides insights into the distribution of distances between scattering entities within the sample, while parameters such as the radius of gyration R_g, maximum particle dimension D_{max}, and chi-squared goodness-of-fit measure the statistical alignment between the experimental data and the proposed model.

Sample 1: GO sample displayed a larger radius of gyration R_g of 122.5 Å, but a slightly smaller maximum particle size D_{max} of 313 Å, compared to the filament. The pair distribution function P(r) showed a prominent peak at 150 Å, similar to the filament, but the overall distribution was more compact, indicative of smaller aggregate structures. This suggests that the GO-modified sample is composed of denser aggregates, likely resulting from the stacking of graphene oxide sheets [21]. The chi-squared value χ^2=0.5816 indicates a good fit, although with more deviation from the model compared to the filament, which could be attributed to the polydispersity and heterogeneous nature of the graphene oxide material. This is consistent with previous studies,

where graphene oxide tends to form compact aggregates in solution [22–24]. The well-behaved residuals and the smooth intensity decay further validate the presence of compact aggregates with reduced long-range order.

Sample 2: mChNF sample showed an intermediate radius of gyration R_g of 118.9 Å and a maximum particle dimension D_{max} of 327 Å. The pair distribution function P(r) exhibited a peak at around 150 Å, with a broad tail extending to 327 Å, indicating the presence of large-scale polymeric or fibrous structures. The higher P(r) error and a more significant Guinier error suggest some uncertainty in the model fit, which might be due to the biopolymeric nature of chitin that forms extended networks. Despite these uncertainties, the chi-squared value χ^2=0.204 remained low, and the residuals were relatively well-accepted. Chitin, being a natural polysaccharide, is known for its fibrillar structure, which is reflected in the GISAXS data. The long tail in the P(r) function is consistent with fibrillar networks, where long-range correlations dominate the scattering behavior [25–29].

Sample 3: Filament, the GISAXS and BIFT analysis revealed a radius of gyration, R_g, of 100.4 Å and a maximum dimension D_{max} of approximately 334 Å. The pair distribution function P(r) exhibits a peak at around 150 Å, indicating a characteristic distance between scattering particles, likely corresponding to the filamentous structure. The long tail in the P(r) distribution suggests the presence of extended structures, which is consistent with the filament morphology. The fit to the experimental scattering data was excellent, as indicated by a low chi-squared value χ^2=0.149, and the residuals were well-behaved. The smooth decay of the intensity I(q) and the long-range correlations observed in P(r) suggest that the filament sample exhibits a network-like structure with significant elongation [30,31].

Comparative Insights: Comparing the three samples, we observe that the GO sample exhibited the largest radius of gyration, followed by chitin and filament. This suggests that the GO sample contains larger or more polydisperse aggregates, while the filament sample is more extended and uniform in terms of particle distribution. The filament and chitin samples share similar long-range correlations, as evidenced by their comparable D_{max} values, but the filament sample appears to be more elongated based on the lower R_g. The chi-squared values for all three samples indicate excellent model fits, with the filament sample providing the best agreement with the experimental data, followed by chitin and GO.

Table S1, SAXS parameters for samples.

Sample	***Chi-Squared (χ^2)***	***D_{max} (Å)***	***R_g (Å)***
Filament	0.149	334.2065	100.4371
GO	0.5816	313.917	122.5334
Chitin	0.204	327.4685	118.8929

The results from this study provide valuable insights into the structural properties of filamentous, graphene oxide-modified, and biopolymeric chitin materials. The BIFT method, combined with GISAXS, allowed for precise real-space reconstructions of the sample structures, highlighting differences in aggregation, long-range order, and overall morphology. These findings contribute to the growing understanding of the nanoscale organization of these materials, which have potential applications in fields ranging from biomaterials to nanocomposites.

2.5.2. GISAXS + GIWAXS Discussion

GO Structural Features: In the GISAXS data for GO (Figure S39a), distinct peaks appear, including a prominent feature at $q = 0.036$ Å^{-1}, corresponding to a d-spacing of ~19 nm. This peak

reflects the regular interlayer spacing within GO sheets, which is characteristic of the material's 2D structure and supports the formation of highly ordered layers. The GIWAXS analysis further confirms this structure, with sharp diffraction peaks at $q = 1.0$ Å^{-1} (d-spacing = 6.7 Å), consistent with the interlayer spacing of GO platelets. These features highlight GO's intrinsic layer-by-layer arrangement, driven by π-π stacking interactions between the GO sheets.

mChNF Structural Features: The GISAXS pattern for mChNF (Figure S39b) reveals broader scattering peaks compared to GO, indicating weaker and more varied packing interactions among chitin nanofibers. A significant peak at $q = 0.026$ Å^{-1} (d-spacing = 24.5 nm) suggests the formation of fibrous aggregates or bundles, pointing to mChNF's semi-crystalline nature. In the GIWAXS region, a distinct peak at $q = 1.36$ Å^{-1} (d-spacing = 4.6 Å) is observed, indicating the presence of crystalline domains within mChNF. This structure results from both ordered crystalline regions and amorphous domains, which coexist in mChNF due to the chitin's partially deacetylated form.

Structural Features of Hybrid GO-mChNF Filaments: The GISAXS and GIWAXS patterns for the hybrid GO-mChNF filaments (Figure 5f) reveal combined features of both GO and mChNF, along with new structural characteristics unique to the hybrid material. The GISAXS pattern shows interlayer distances ranging from 15.7 Å to 122.7 Å, suggesting a mixed organization of GO sheets and mChNF fibers. This combination is marked by reduced form factor oscillations, indicating a more disordered assembly due to the interfacial jamming of nanoparticles. In the GIWAXS data, new peaks at $q = 1.43$ Å^{-1} and $q = 2.0$ Å^{-1} appear, likely due to electrostatic complexation between negatively charged GO and positively charged amine groups on mChNF. These interactions contribute to the formation of novel crystalline features in the hybrid, demonstrating that electrostatic attraction and π-π stacking lead to a rearranged structure. The shift in peak positions

and intensities in both GISAXS and GIWAXS data suggests a denser, more stable material, capitalizing on the properties of both GO and mChNF.

Azimuthal Intensity Profiles: Azimuthal intensity profiles provide additional insight into the alignment and isotropy within mChNF and hybrid filament samples (Figure S40). In the case of mChNF alone (Figure S40a), the profile shows a sharp peak centered around 270°, with a high alignment parameter ($R \sim 0.75$). This suggests a well-organized crystalline domain structure, likely due to the effects of evaporation on the 1-butanol bath during testing. In contrast, the azimuthal profile for the hybrid filament sample (Figure S40b) is broader, with reduced alignment ($R \sim 0.15$), indicating a more isotropic arrangement. This decreased alignment reflects the impact of rapid interfacial self-assembly, which disrupts the crystallinity of mChNF during complexation with GO, resulting in a less ordered structure in the hybrid filaments.

3. Supporting Figures

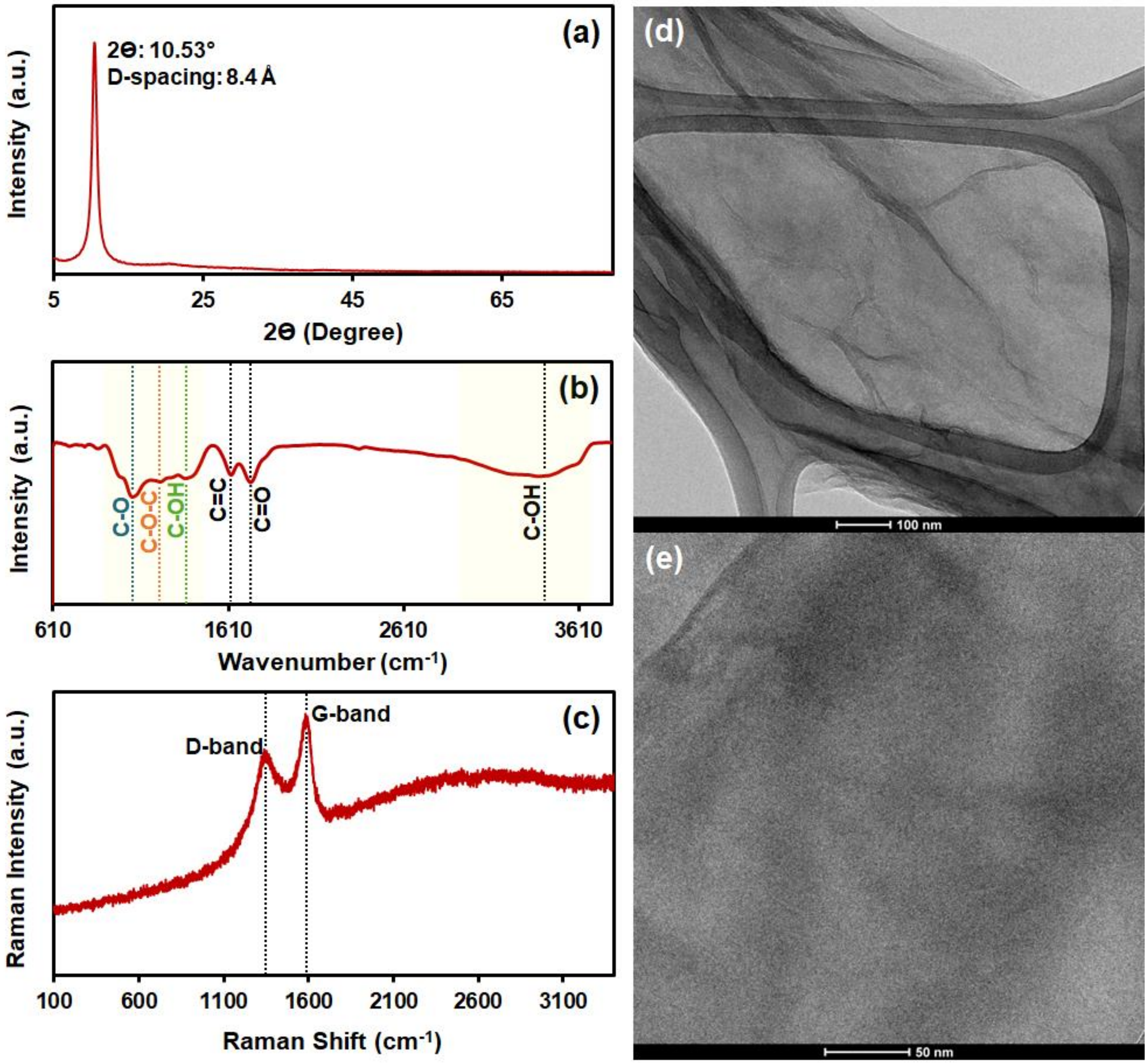

Figure S1. Characterization of GO, including (a) X-ray diffraction, (b) FTIR, (c) micro-Raman spectroscopy, and (d-e) HRTEM analysis.

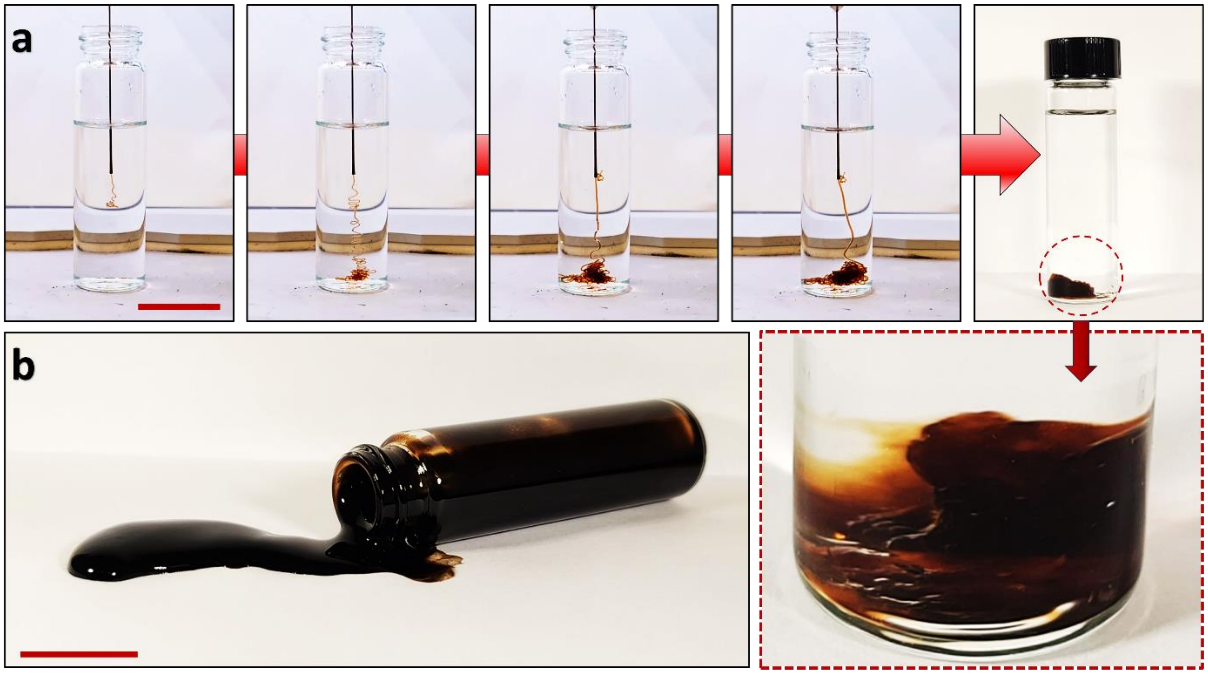

Figure S2. (a) a sequence of images showing the streaming of aqueous GO (10 mg/ml) into 1-butanol, illustrating that the stream structure is not stable upon contact with the solvent for a while. (b) Demonstration of the flowability of GO. Scale bars in (a) and (b) correspond to 3 and 2.5 cm.

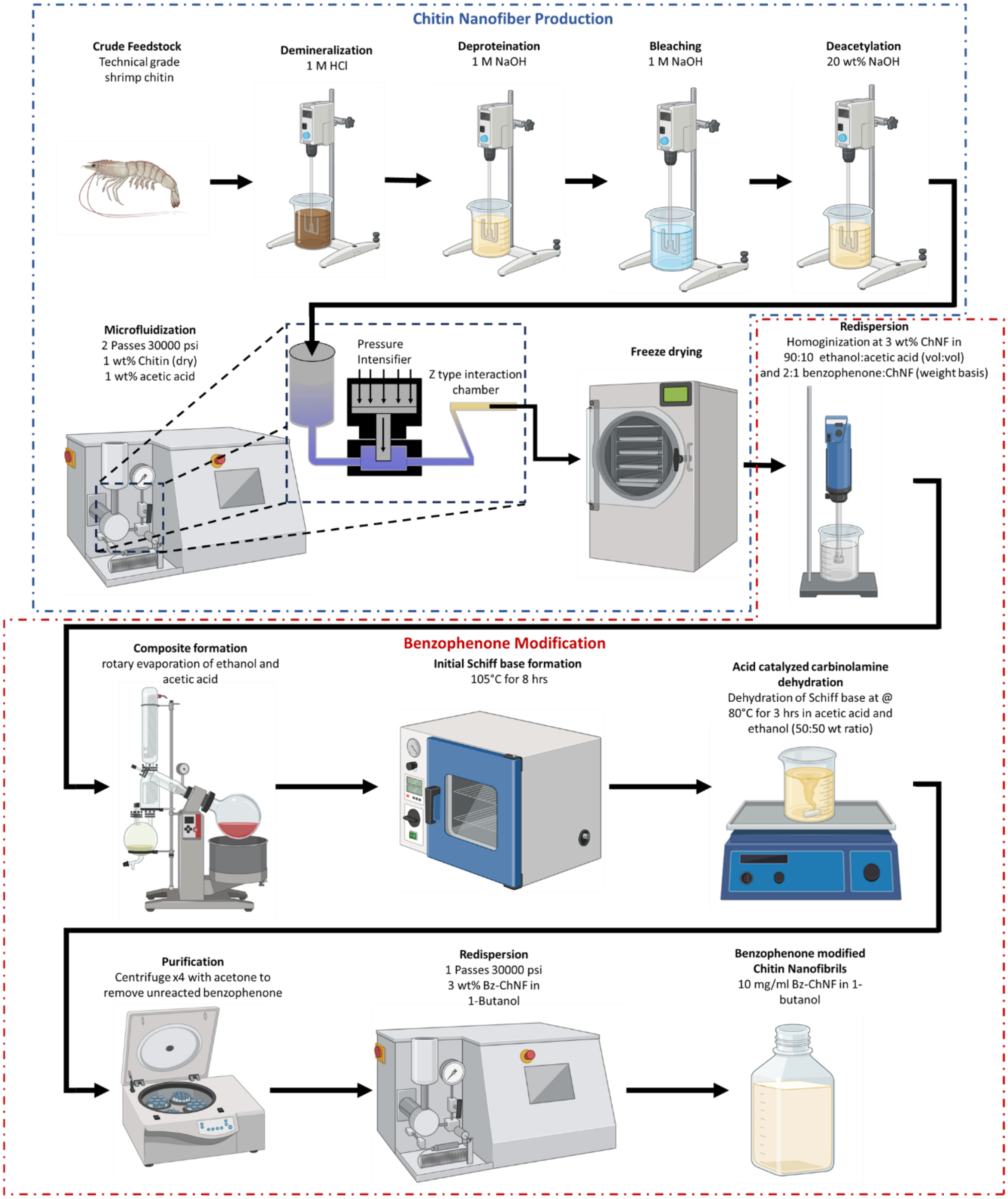

Figure S3. Schematic representation of the experimental processes for Chitin Nanofiber Production and Benzophenone Modification. The chitin nanofiber production process (top section) involves the mechanical defibrillation of chitin into nanofibers, freeze-drying, and sonication. The benzophenone modification (bottom section) shows the preparation steps including mixing, drying, centrifugation, and extrusion to achieve modified chitin nanofibers.

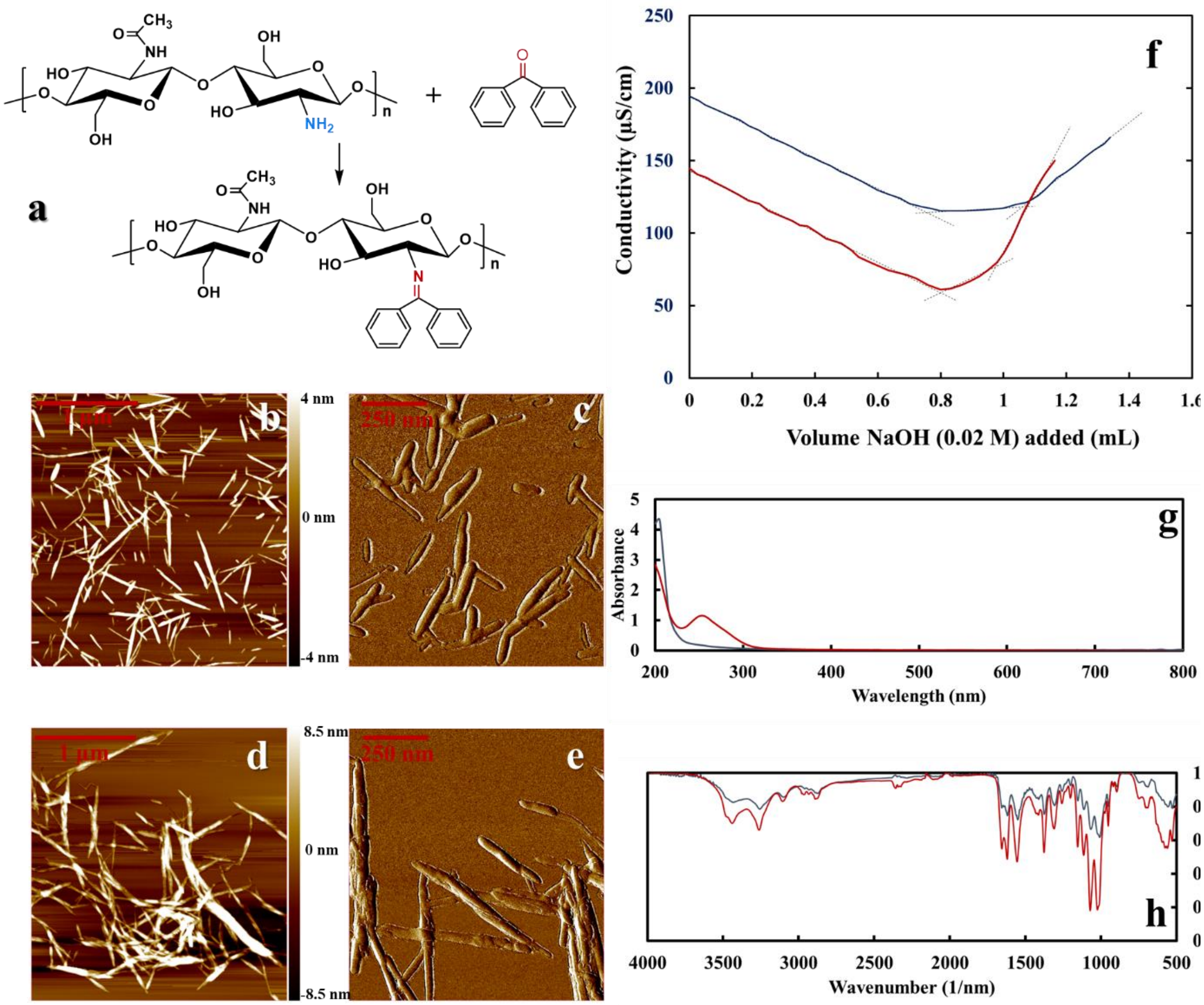

Figure S4. Characterization of modified chitin nanofibers. a) Chemical modification performed on the primary amine groups. b-e) AFM measurements of the unmodified (b&c) and modified (d&e) chitin nanofibers. f) Conductometric titration of unmodified (blue) and modified (red) chitin nanofibers. g) UV-Vis of unmodified (blue) and modified (red) chitin nanofibers. h) FTIR of unmodified (blue) and modified (red) chitin nanofibers.

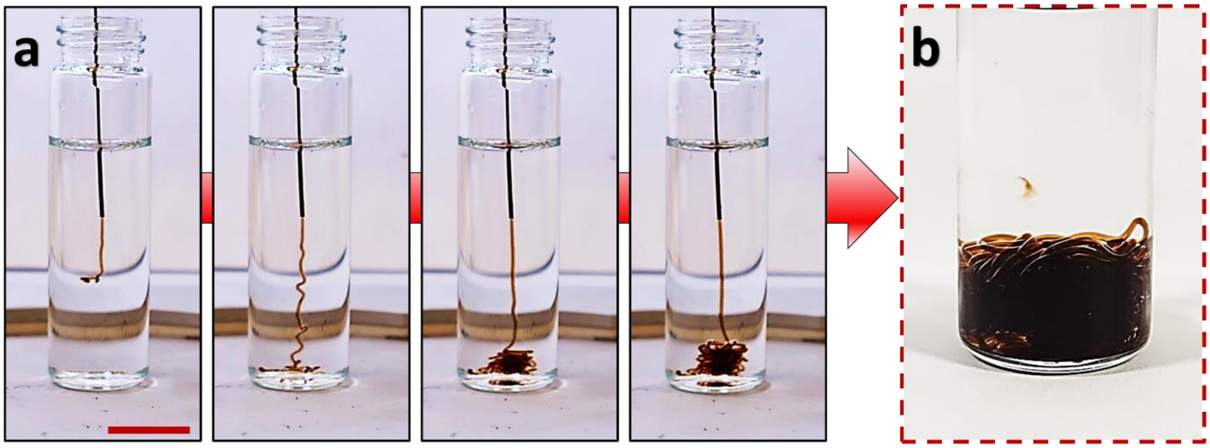

Figure S5. (a) a sequence of images showing the streaming of aqueous GO (10 mg/ml) into mChNF/1-butanol (1 mg/ml), (b) illustrating that the stream structure is stable due to interfacial intraction. Scale bars in (a) correspond to 2 cm.

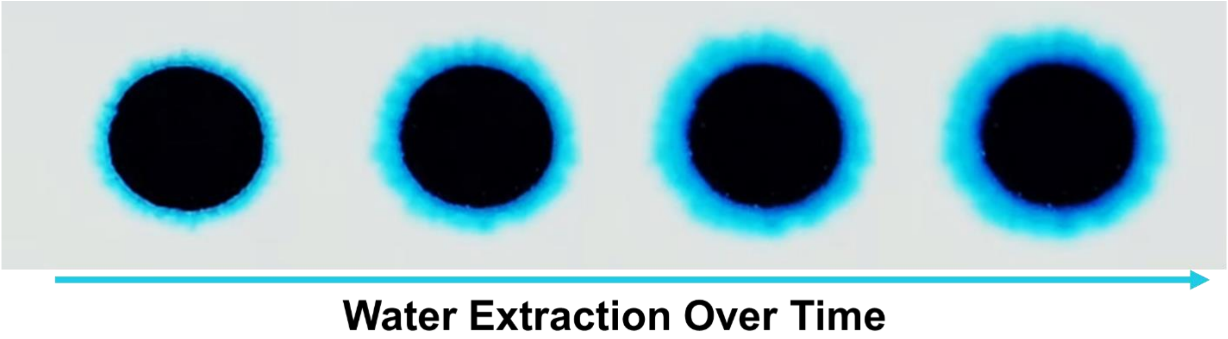

Figure S6. Permeable interface between GO-ink droplets printed into a 1 mg/mL modified mChNF bath, showing gradual diffusion at the interface.

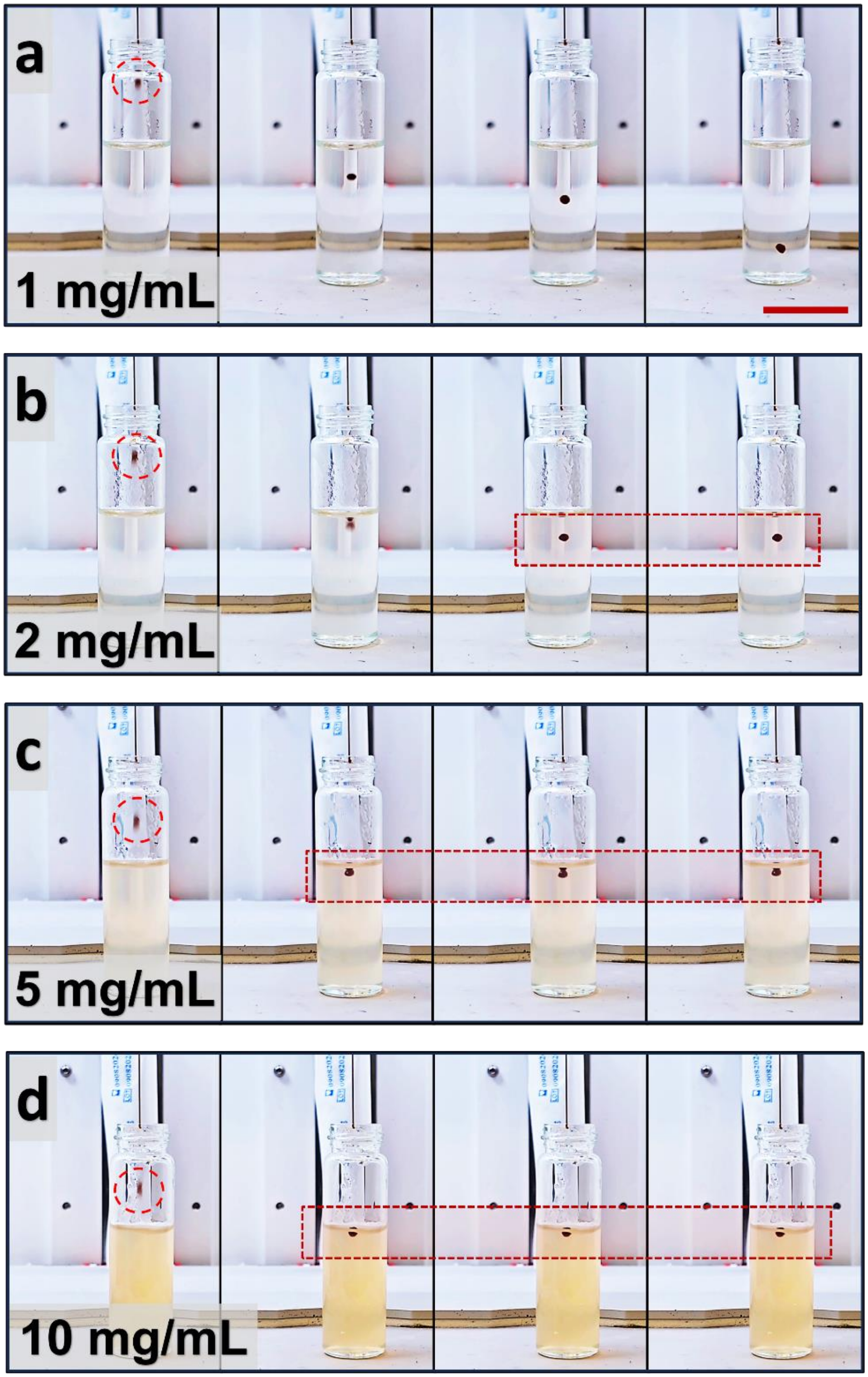

Figure S7. Visualization of GO droplet into 1-butanol at varying concentrations of mChNF: (a) 1 mg/mL, (b) 2 mg/mL, (c) 5 mg/mL, and (d) 10 mg/mL. The progression of GO behavior is shown for each concentration as it enters the bath, illustrating the differences in settling behavior and interaction with the solvent across the concentration range. Scale bar correspond to 3.3 cm.

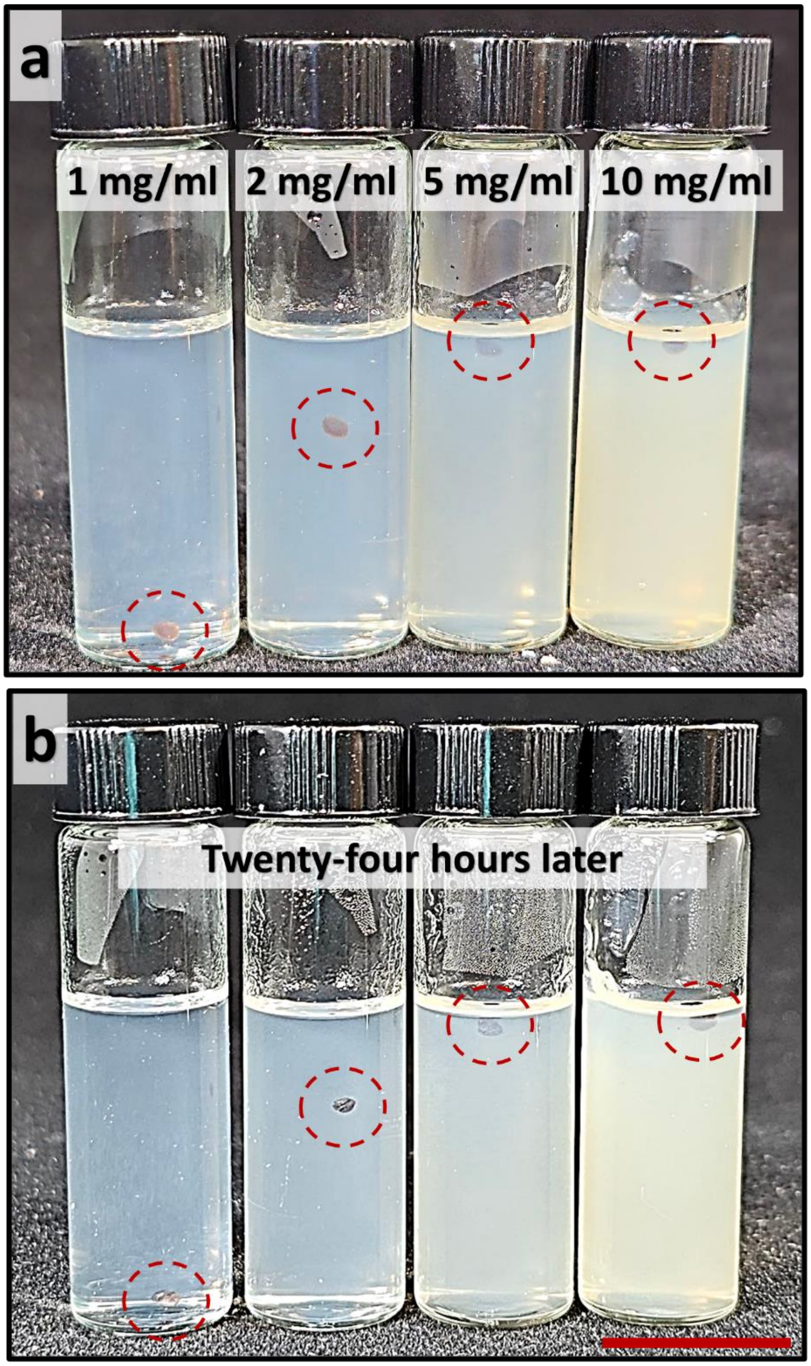

Figure S8. Stability behavior of GO droplet in 1-butanol at different concentrations of mChNF: (a) Immediately after injection with concentrations ranging from 1 mg/mL to 10 mg/mL, and (b) after 24 hours. Scale bars correspond to 3.9 cm.

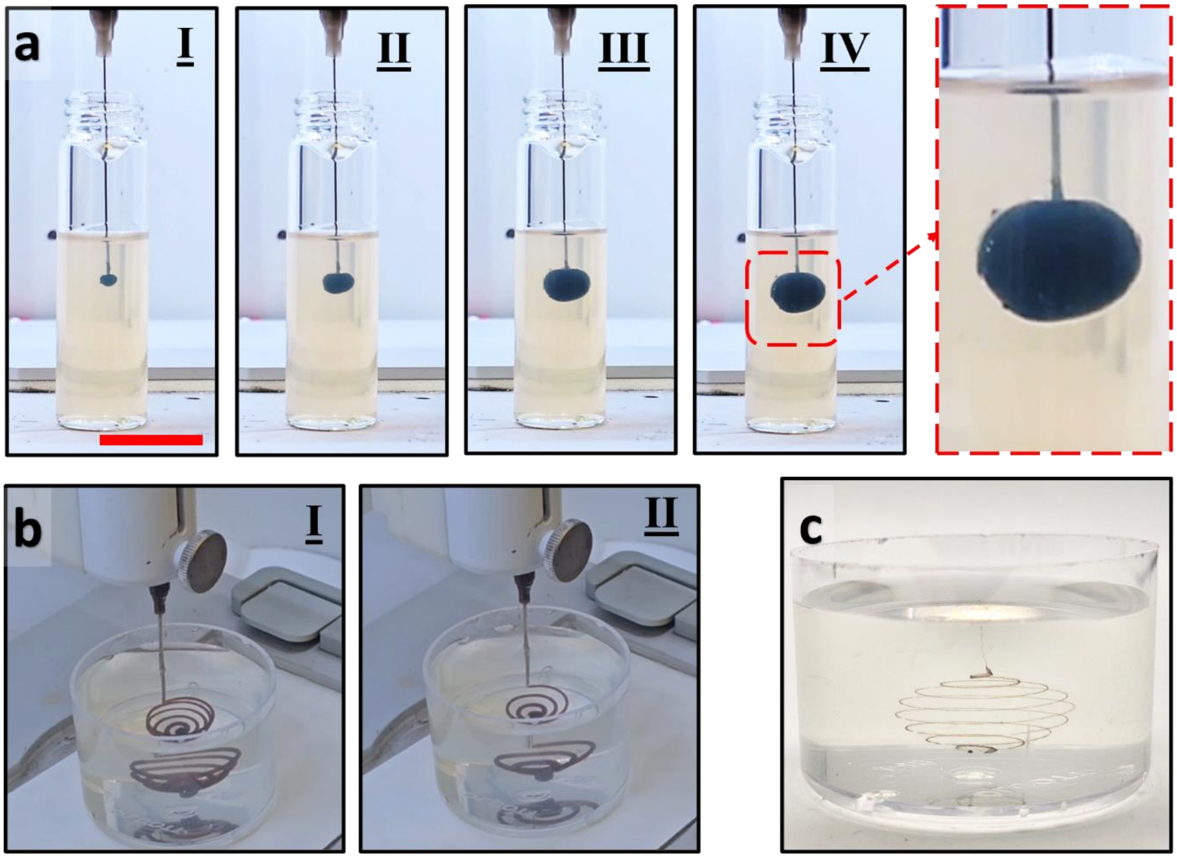

Figure S9. (a) The 1-butanol external phase with 10 mg/mL mChNF shows gel-like properties, allowing a large droplet of 10 mg/mL GO suspension to be pinned in place. Scale bar = 2.4 cm. Spatial liquid-in-liquid printing of a 10 mg/mL GO aqueous suspension using a 16-gauge (b) and 27-gauge (c) needle into 5 mg/mL mChNF/1-butanol.

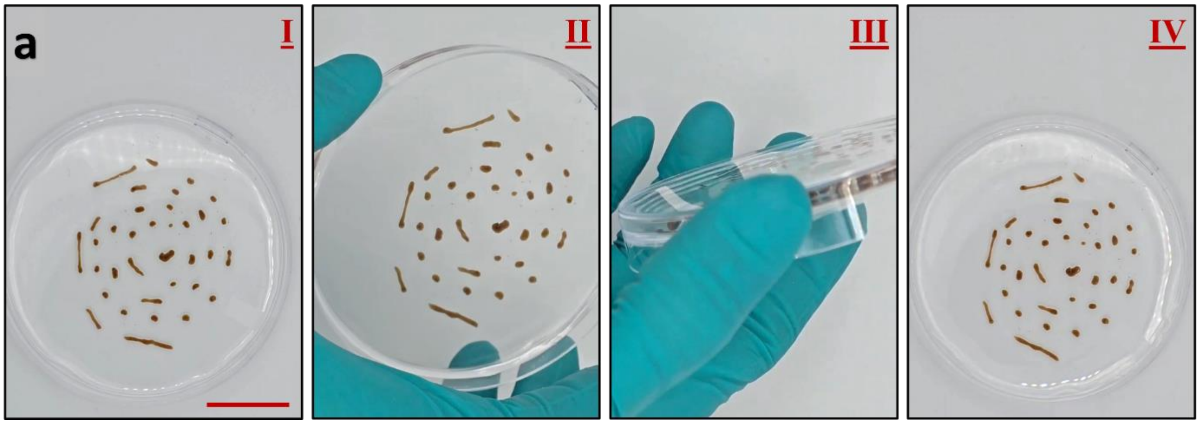

Figure S10. Printed GO on a highly viscous silicone oil bath. (I-IV) Sequential views highlight the disability of GO writing due to the absence of interfacial interaction. Scale bars correspond to 4 cm.

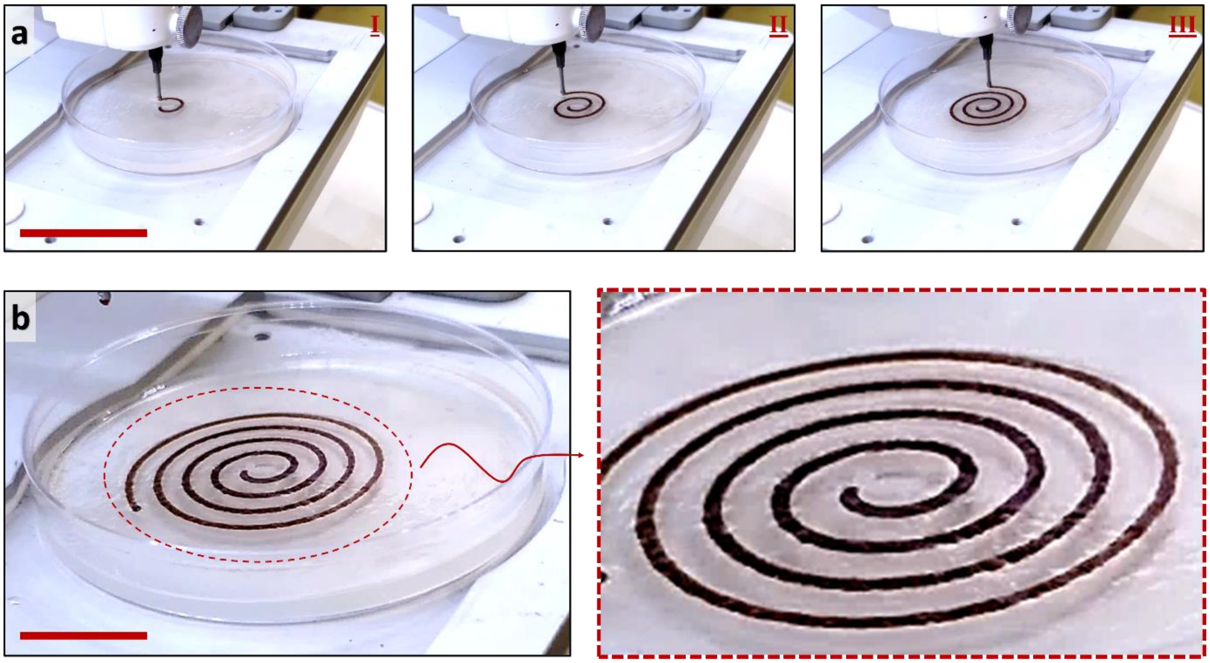

Figure S11. (a) Sequential printing process of a spiral pattern with gauge-27 of GO ink in 5 mg/ml mChNF bath (I-III). (b) A closer view of the completed spiral structure shows uniformity and precision in printing. Scale bars in (a) and (b) correspond to 5.5 cm and 2.9 cm.

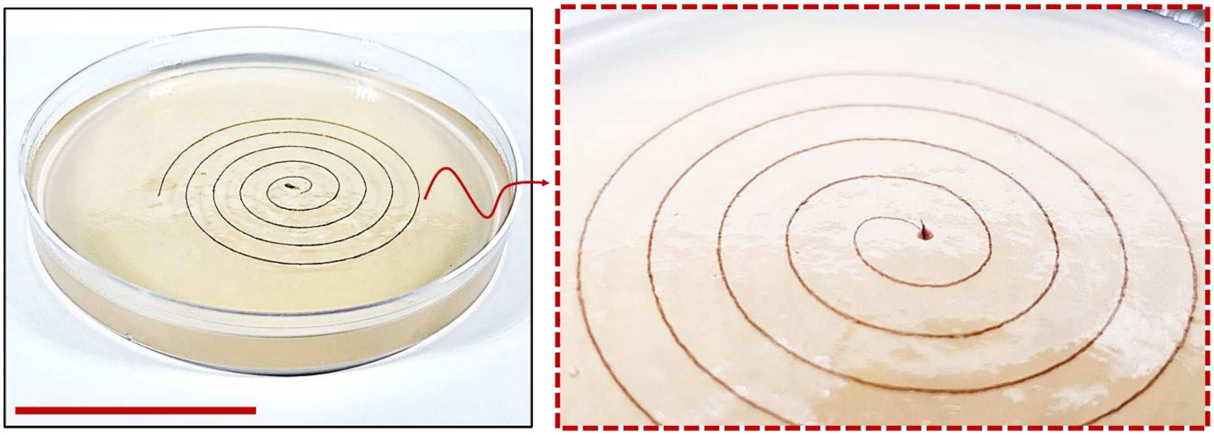

Figure S12. A closer view of the completed spiral pattern with gauge-16 of GO ink in 10 mg/ml mChNF bath. Scale bars correspond to 4.6 cm.

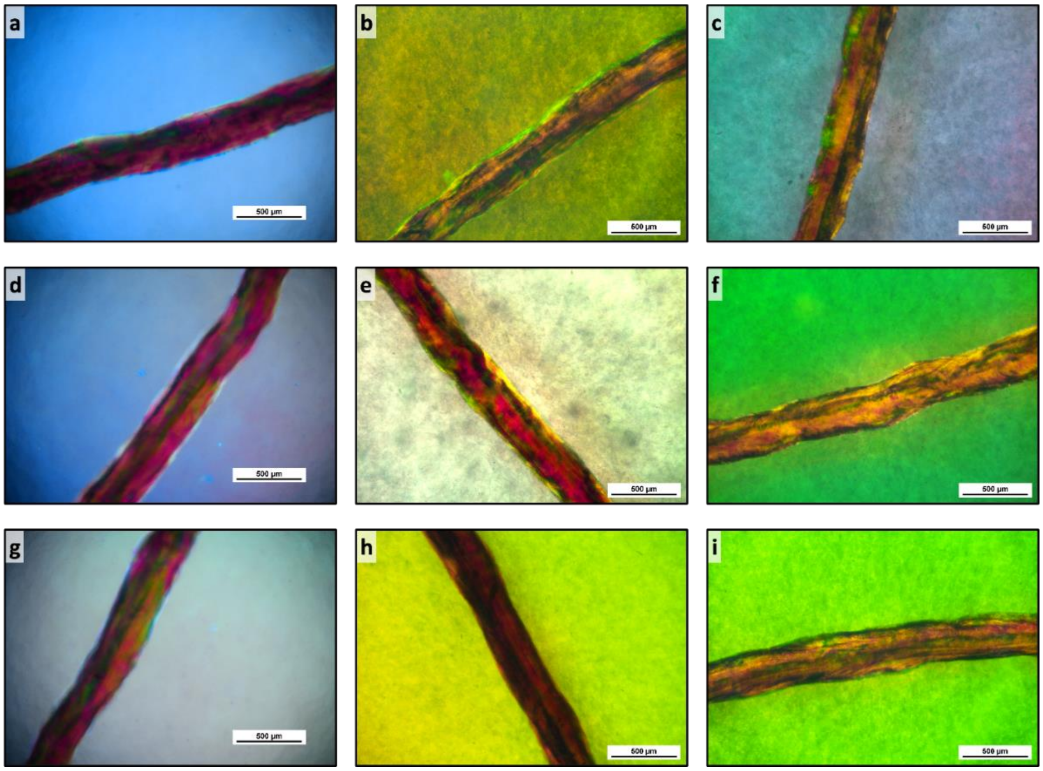

Figure S13. Optical microscopy images of GO-ink printed into mChNF baths using a gauge 27 needle, demonstrating the structural consistency of the filaments. Images (a-i) show the printed liquid, emphasizing surface morphology and uniformity.

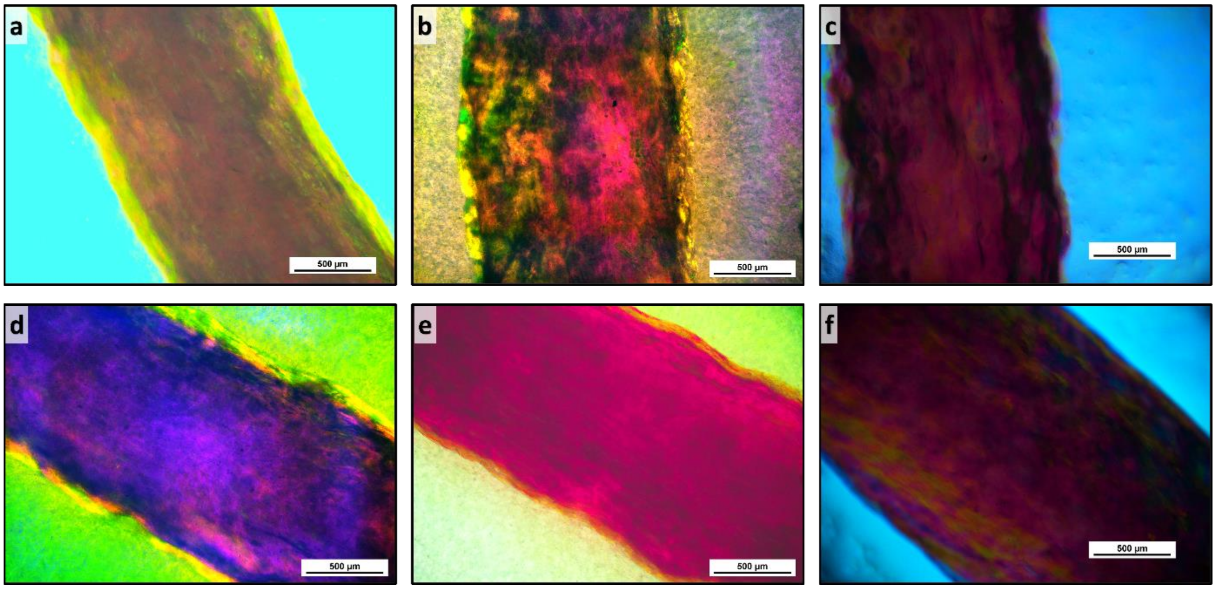

Figure S14. Optical microscopy images of GO-ink printed into mChNF baths using a gauge 16 needle, demonstrating the structural consistency of the filaments. Images (a-i) show the printed liquid, emphasizing surface morphology and uniformity.

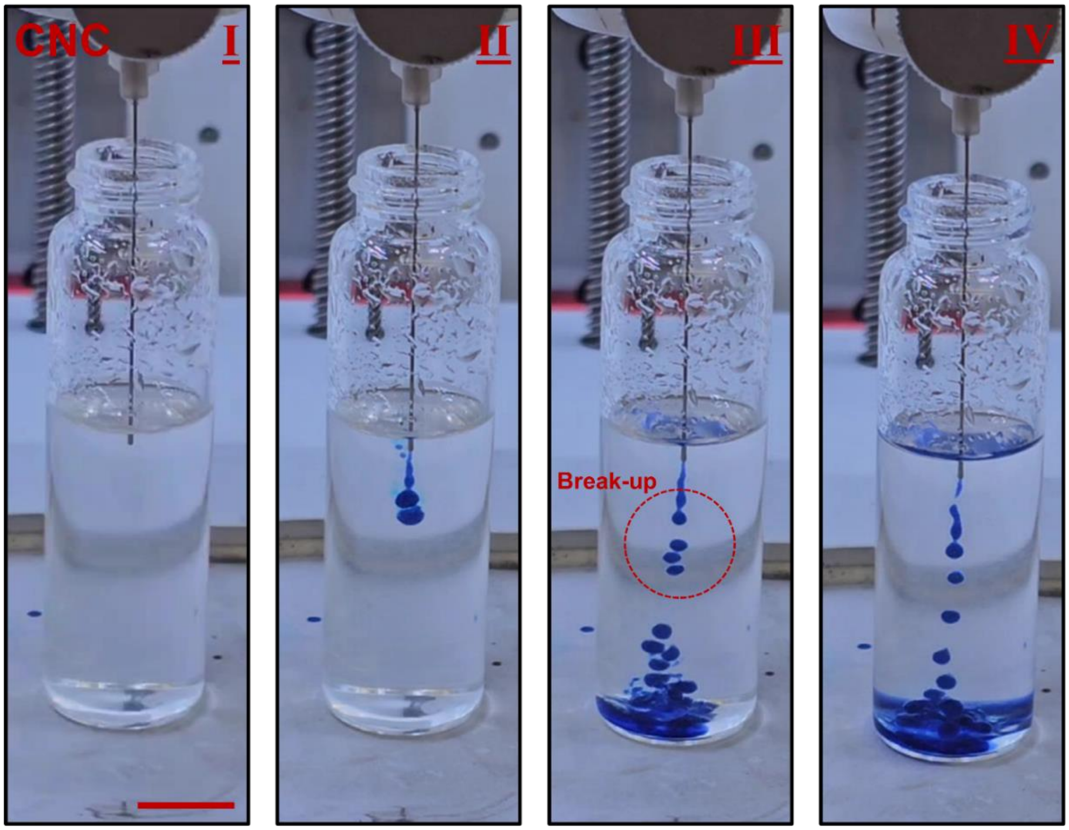

Figure S15. Images showing the printing process of CNC ink into an mChNF bath (1 mg/mL). Due to the absence of strong interfacial interactions, the CNC ink cannot form a stable printed structure, leading to break-up as shown in the circled region in frame III. This demonstrates that CNC ink in this system is not suitable for printing. Scale bar correspond to 1.9 cm.

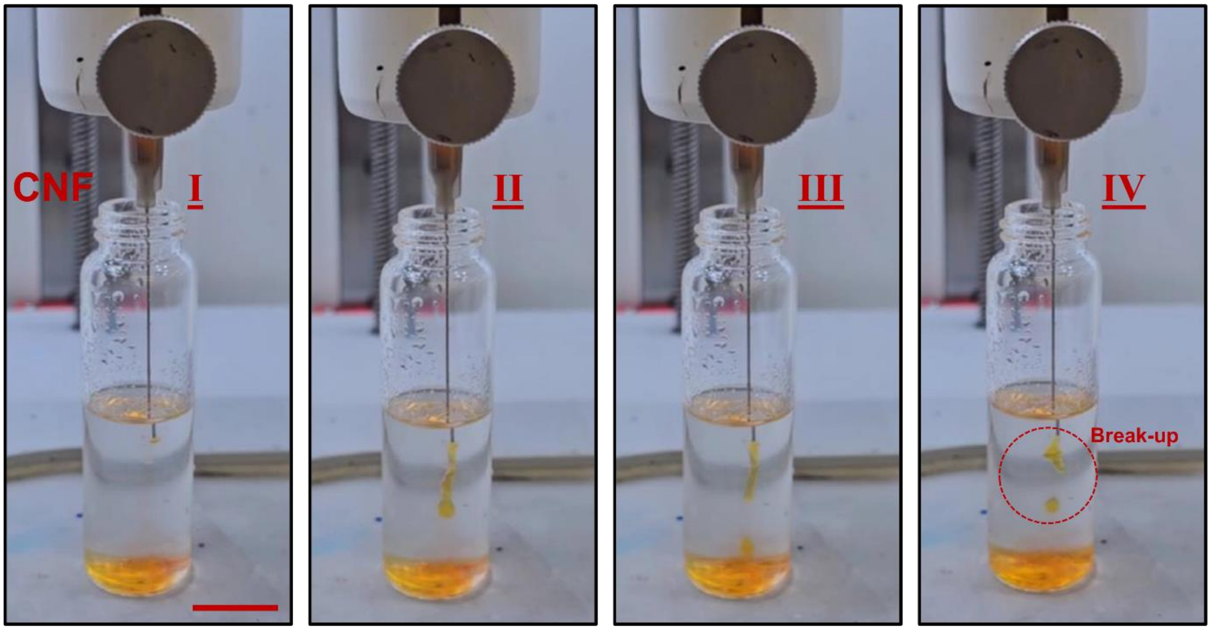

Figure S16. Images showing the printing process of CNF ink into an mChNF bath (1 mg/mL). Due to the absence of strong interfacial interactions, the CNF ink cannot form a stable printed structure, leading to break-up as shown in the circled region in frame IV. This demonstrates that CNF ink in this system is not suitable for printing. Scale bar correspond to 2.1 cm.

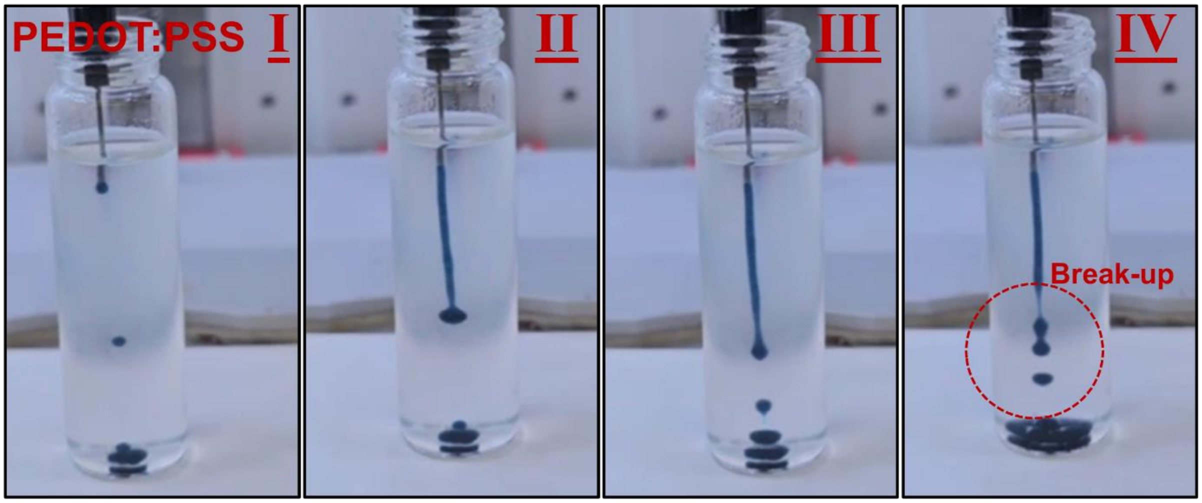

Figure S17. Images showing the printing process of PEDOT:PSS ink into an mChNF bath (1 mg/mL). Due to the absence of strong interfacial interactions, the PEDOT:PSS ink cannot form a stable printed structure, leading to break-up as shown in the circled region in frame IV. This demonstrates that PEDOT:PSS ink in this system is not suitable for printing.

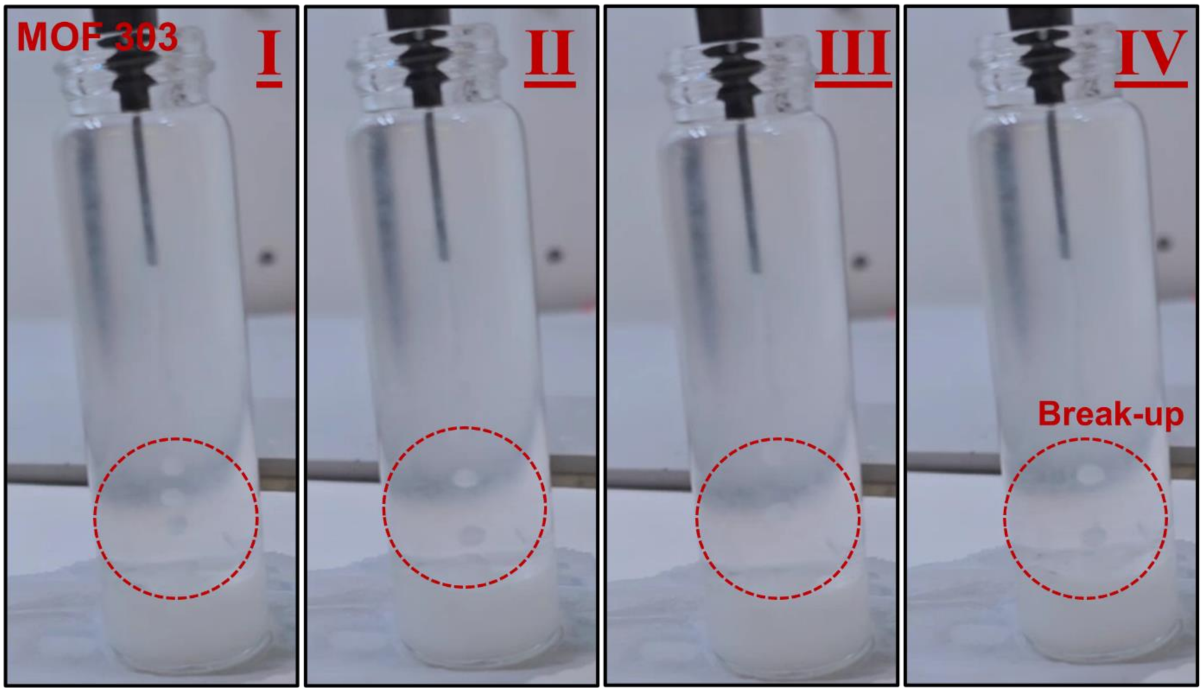

Figure S18. Images showing the printing process of MOF-303 ink into an mChNF bath (1 mg/mL). Due to the absence of strong interfacial interactions, the MOF 303 ink cannot form a stable printed structure, leading to break-up as shown in the circled region in frame IV. This demonstrates that MOF 303 ink in this system is not suitable for printing.

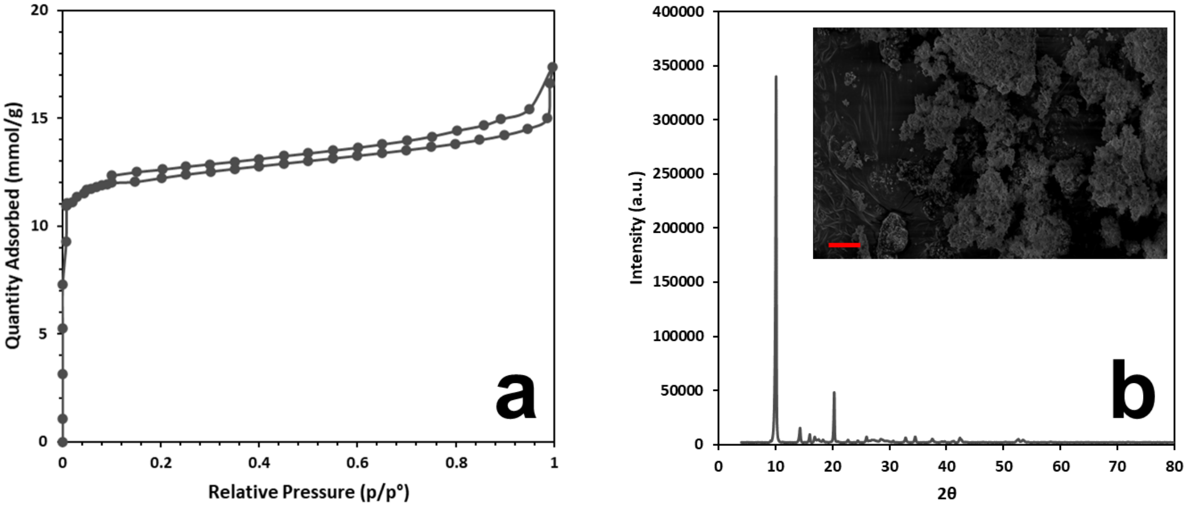

Figure S19. (a) Adsorption-desorption isotherms, showing the specific surface area and pore size distribution of the material. (b) X-ray diffraction (XRD) pattern indicating the crystalline structure of MOF303 with peaks corresponding to specific crystallographic planes and (inside) SEM image of powder (scale bar=10 μm).

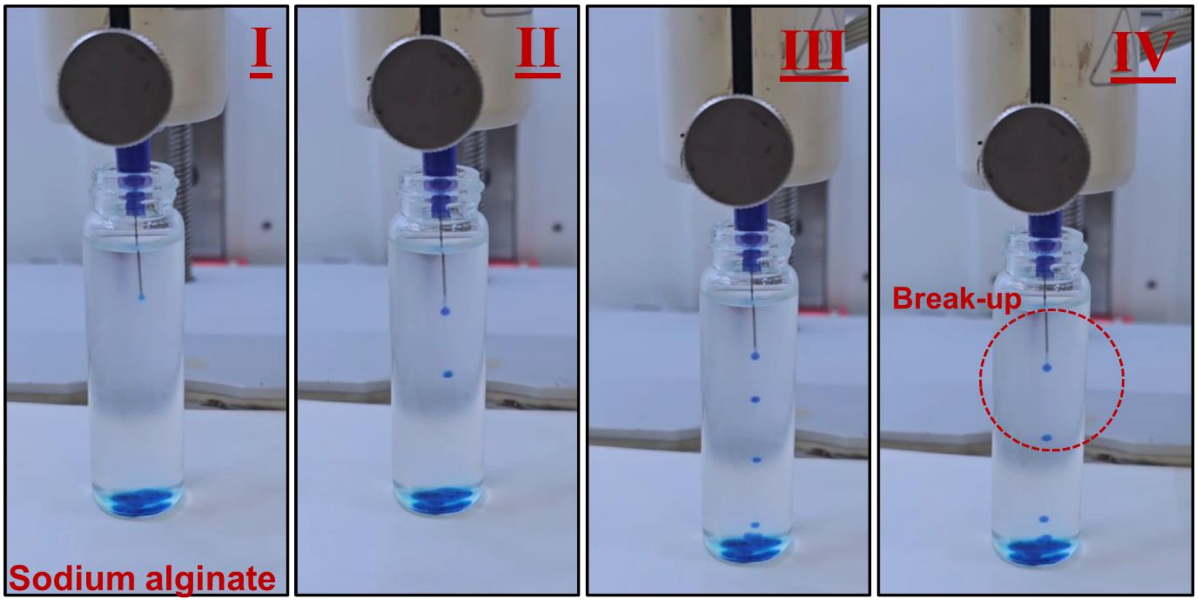

Figure S20. Images showing the printing process of Sodium Alginate (SA) ink into an mChNF bath (1 mg/mL). Due to the absence of strong interfacial interactions, the SA ink cannot form a stable printed structure, leading to break-up as shown in the circled region in frame IV. This demonstrates that SA ink in this system is not suitable for printing.

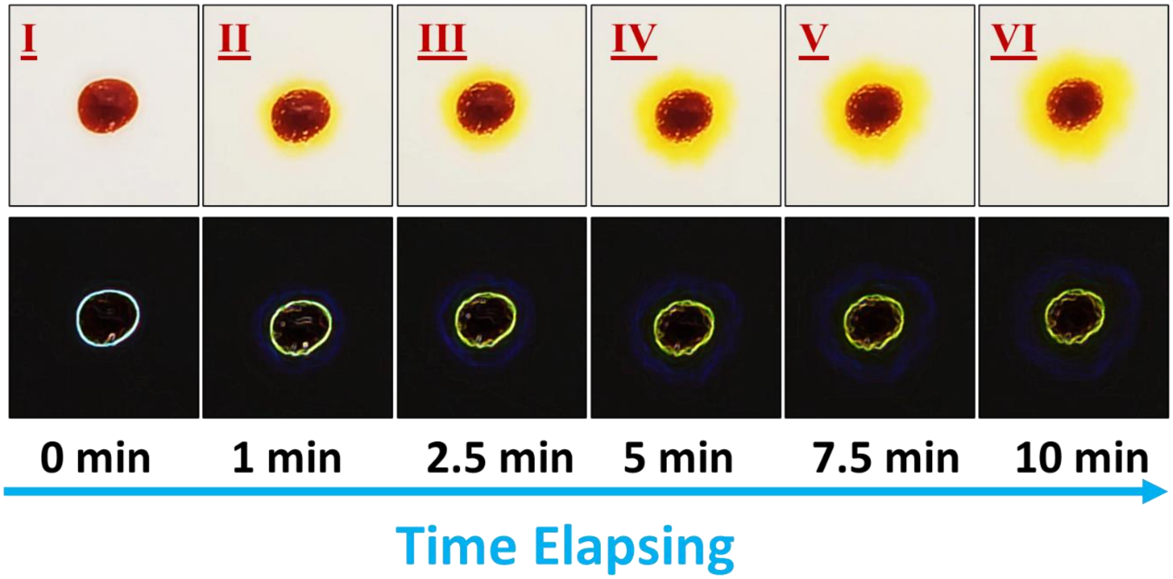

Figure S21. Permeable interface between GO+NaAlg-ink droplets printed into a 1 mg/mL modified mChNF bath, showing gradual diffusion at the interface. The top row shows the original images of the droplet over time, while the second row displays processed images enhancing the interface clarity to emphasize the diffusion pattern.

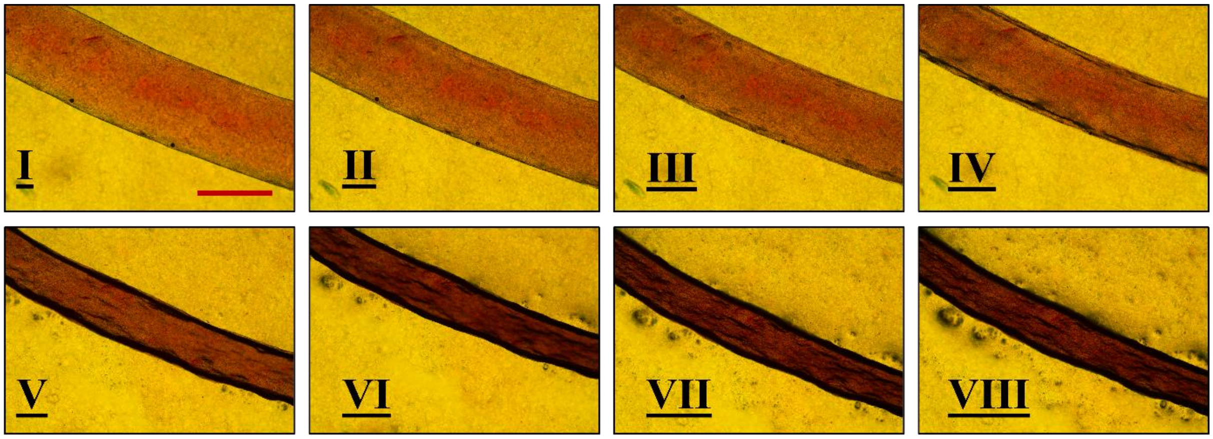

Figure S22. The optical microscope images of a printed line showcase water removal and solidification of GO/sodium alginate filaments. Pictures (I), (II), (III), and (IV) were captured at x, y, z, and t minutes after printing, respectively, highlighting the gradual solidification process. Scale bars in (a) and (b) correspond to 250 µm.

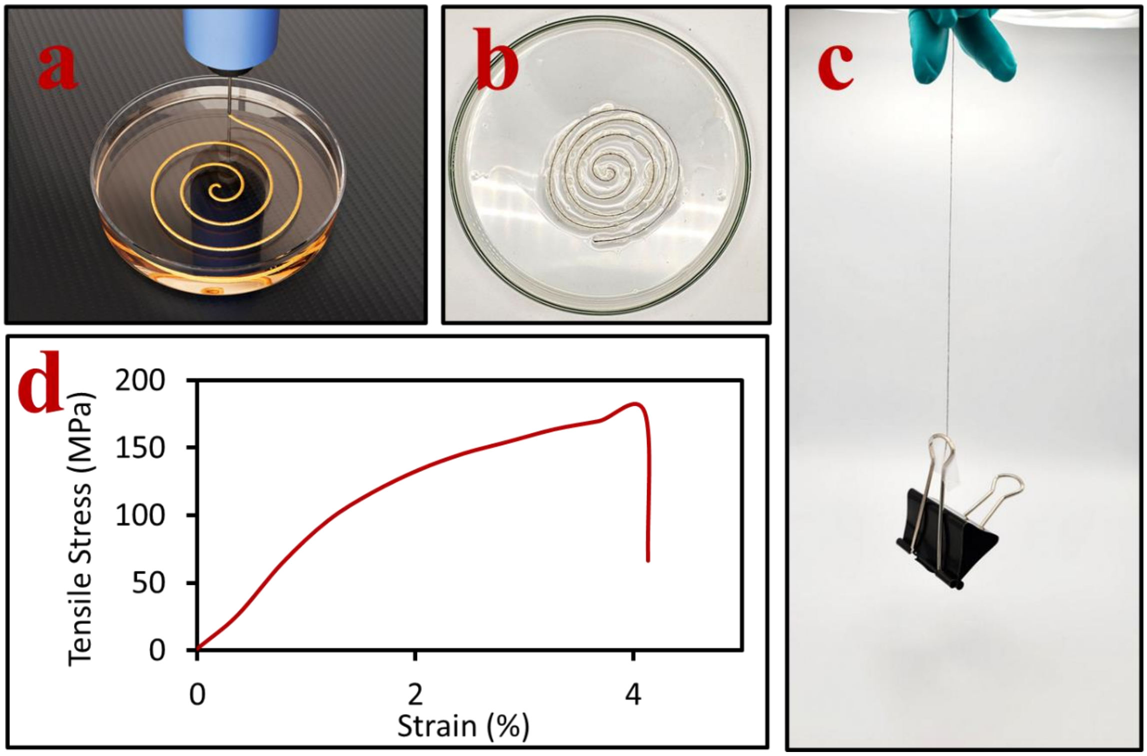

Figure S23. Schematic illustration of printing an aqueous GO/sodium alginate suspension into the mChNF/1-butanol bath. (b) The printed spiral structure immediately after water removal and solidification in the mChNF/1-butanol bath. (c) The solidified filament showing mechanical stability by supporting a weight of 25 g after removal from the bath and drying at atmospheric conditions. (d) The stress-strain curve of the GO/sodium alginate filament, demonstrating the enhanced mechanical properties attributed to the interfacial assembly of GO and mChNF. The GO/sodium alginate ink had a total solid concentration of 5.5 wt% with a sodium alginate to GO ratio of 10:1, printed in an mChNF/1-butanol bath at 5 mg/mL.

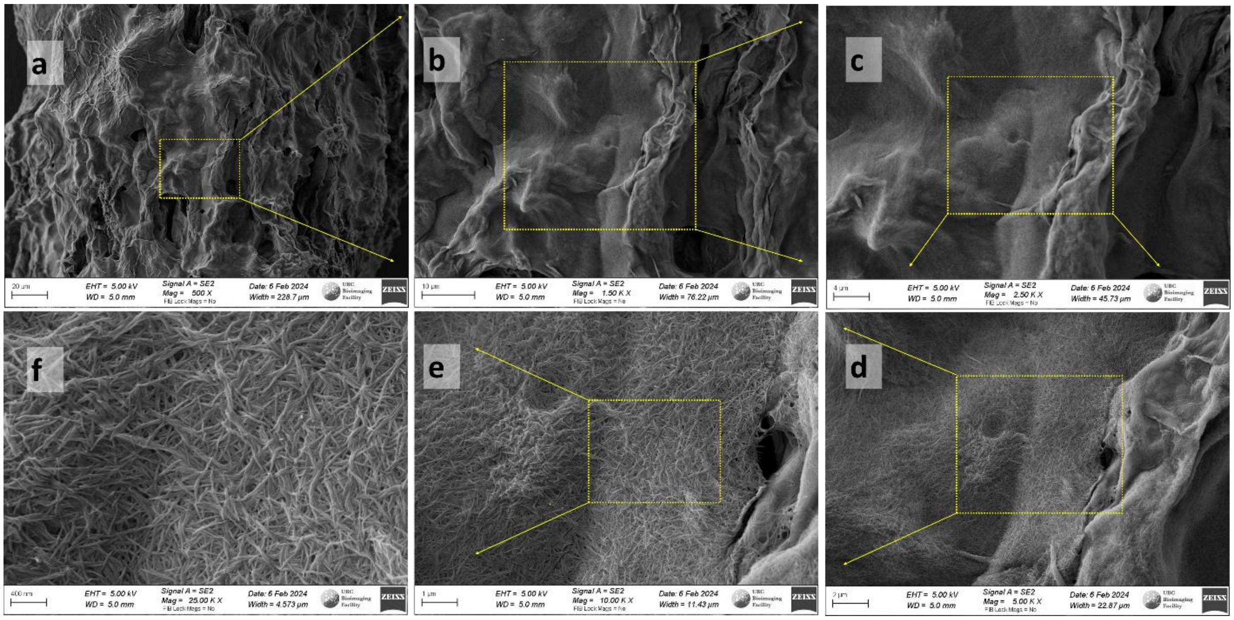

Figure S24. SEM images highlighting the surface of GO/sodium alginate filaments. Images (a-f) show detailed surface textures and reveal the presence of mChNFs within the skin layers of the filaments. The yellow boxes indicate areas magnified to showcase the intricate structure of the filaments at different scales, emphasizing the interfacial assembly between GO and mChNFs. Scale bars: (a) 20 µm, (b) 10 µm, (c) 5 µm, (d) 3 µm, (e) 1 µm, and (f) 400 nm.

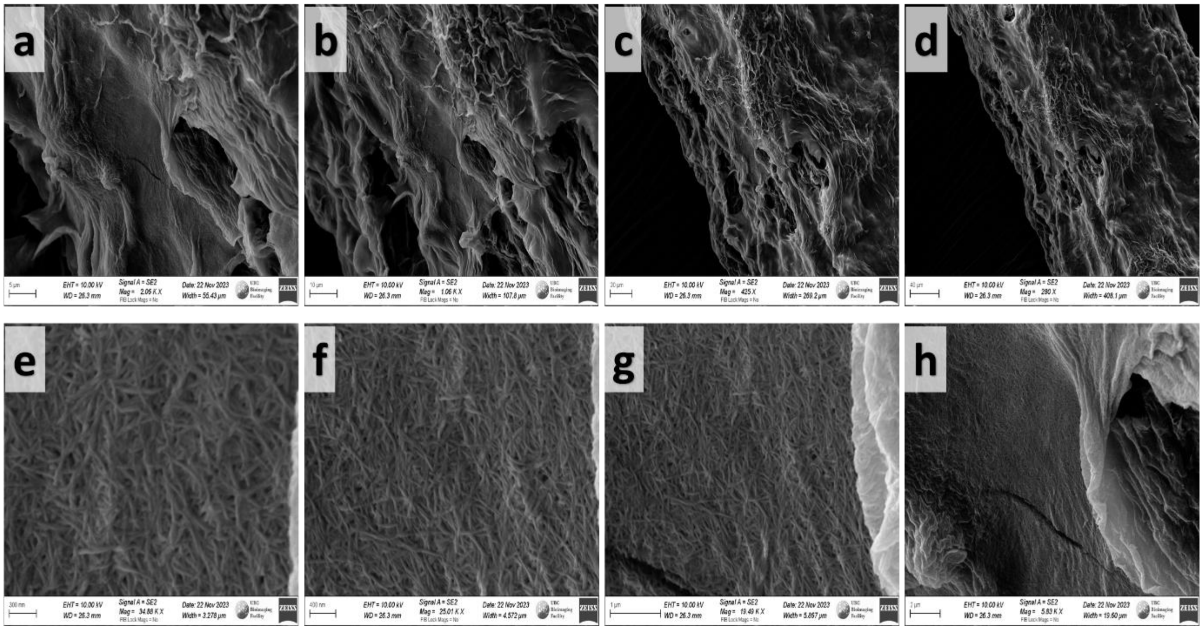

Figure S25. SEM images showing the surface morphology of the GO/sodium alginate filaments. The images (a-h) highlight the rough surface texture due to the presence of numerous mChNFs pinned at the interface, resulting from the assembly of GO-mChNF nanoparticles. The surface exhibits a dense, interfacially assembled layer, contributing to the mechanical stability of the filaments.

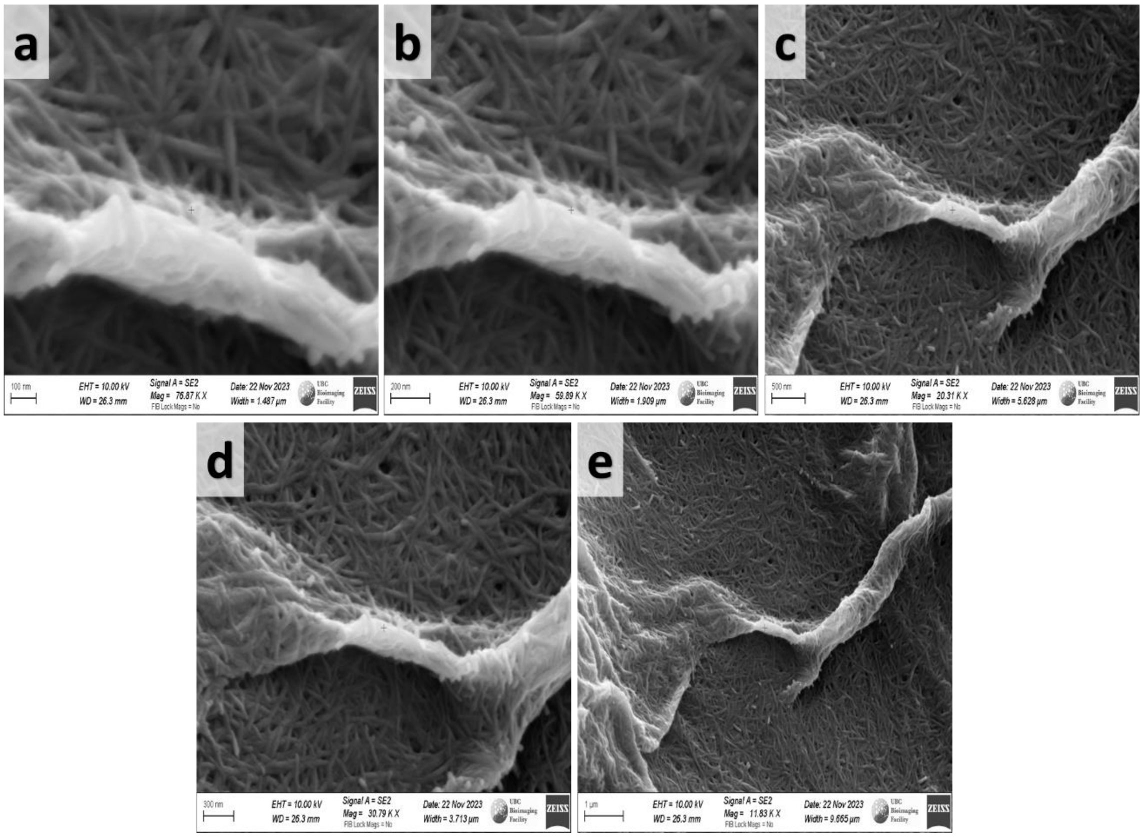

Figure S26. The images (a-e) reveal a skin morphology, where the outer layer (skin) is densely packed with mChNFs. This structure results from the jamming of GO and mChNFs at the interface during filament formation.

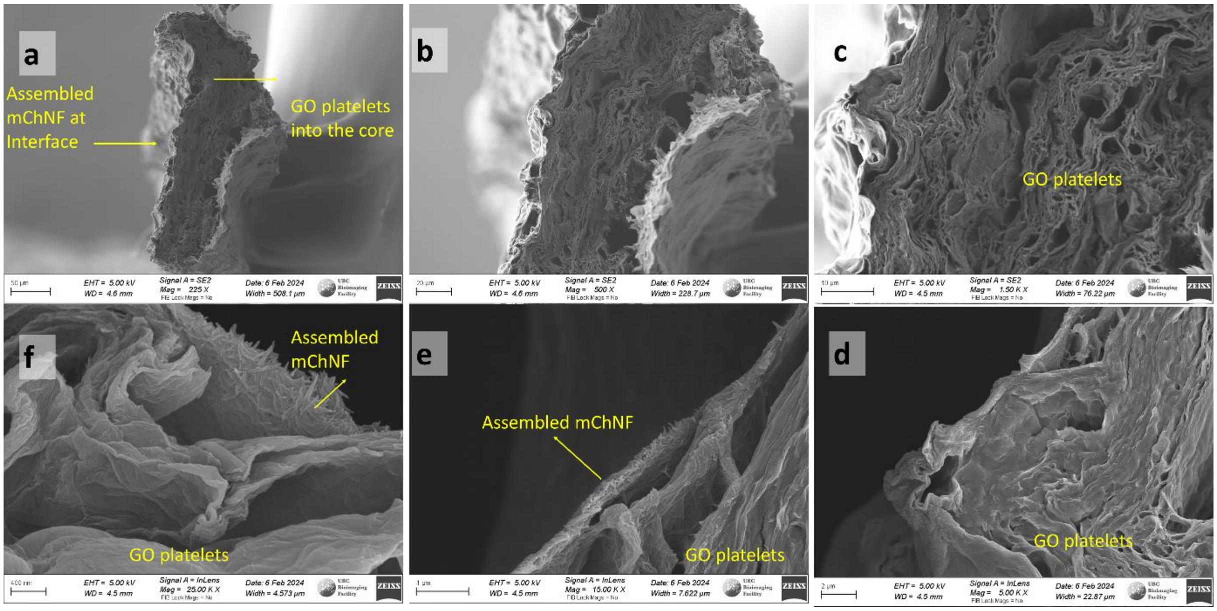

Figure S27. SEM images illustrate the cross-sectional view of GO/sodium alginate filaments, showcasing the assembly of mChNFs at the interface and the distribution of GO platelets into the core. The yellow labels indicate the presence of mChNFs and GO platelets at various points in the filament structure. Scale bars: (a) 10 µm, (b) 2 µm, (c) 1 µm, (d) 3 µm, (e) 5 µm, and (f) 400 nm.

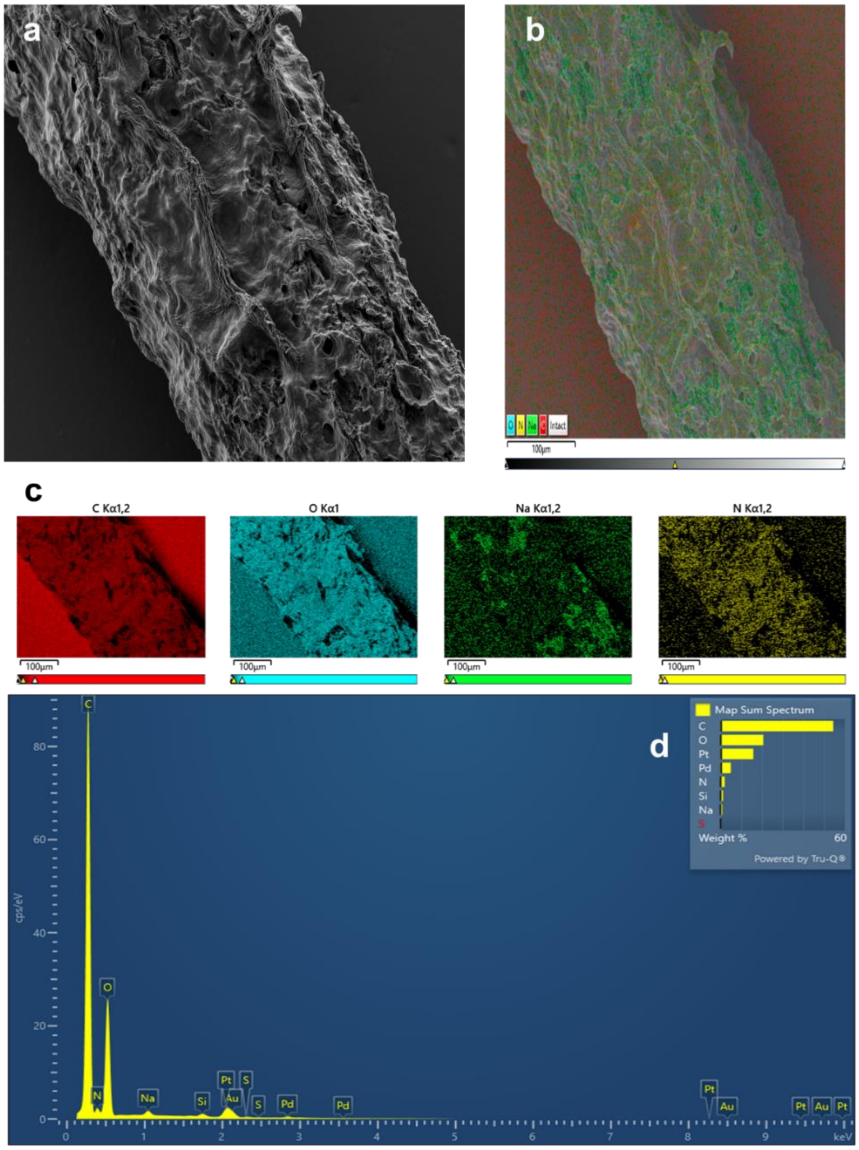

Figure S28. (a) SEM image of the GO/sodium alginate filament showing surface morphology, (b) EDS overlay mapping for C, O, Na, and N showing the elemental distribution across the filament, (c) individual EDS maps for C, O, N, and Na demonstrating the distribution of these elements on the filament's surface, and (d) corresponding EDS spectrum, showing the relative elemental composition.

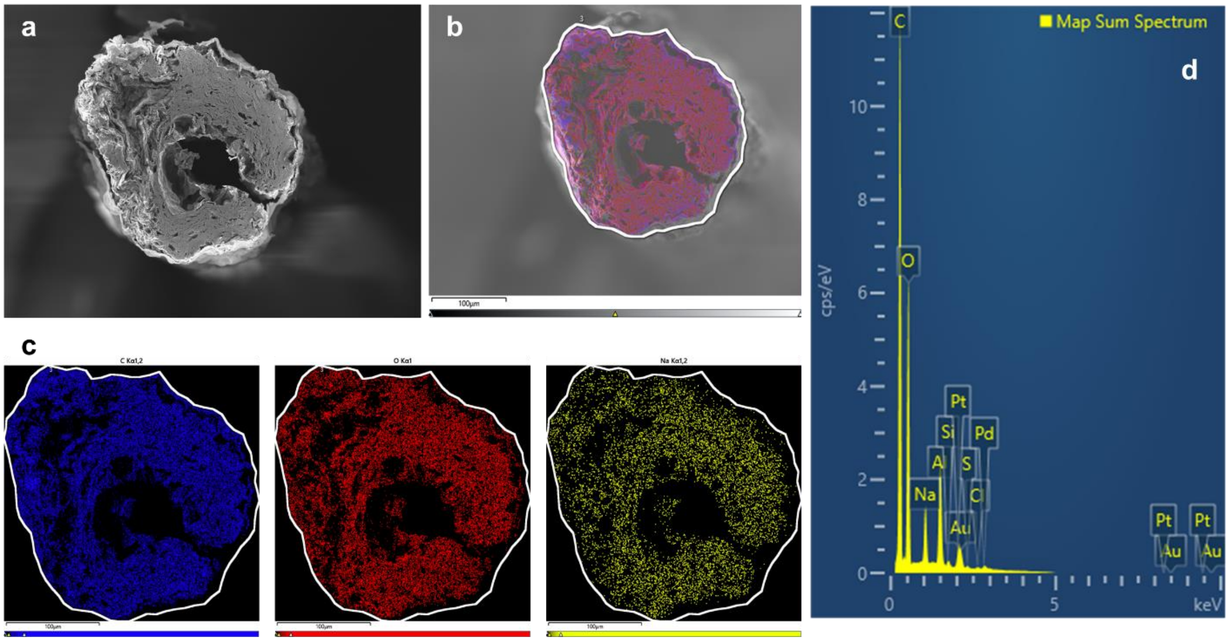

Figure S29. (a) Cross-sectional SEM image of a GO/sodium alginate filament, (b) EDS mapping overlay showing the distribution of carbon (blue), oxygen (red), and sodium (yellow) in the filament, and (c) individual EDS maps of carbon, oxygen, and sodium elements. (d) Corresponding EDS spectrum, confirming the presence of C, O, Na, and other trace elements. The scale bar in (b) represents 100 µm.

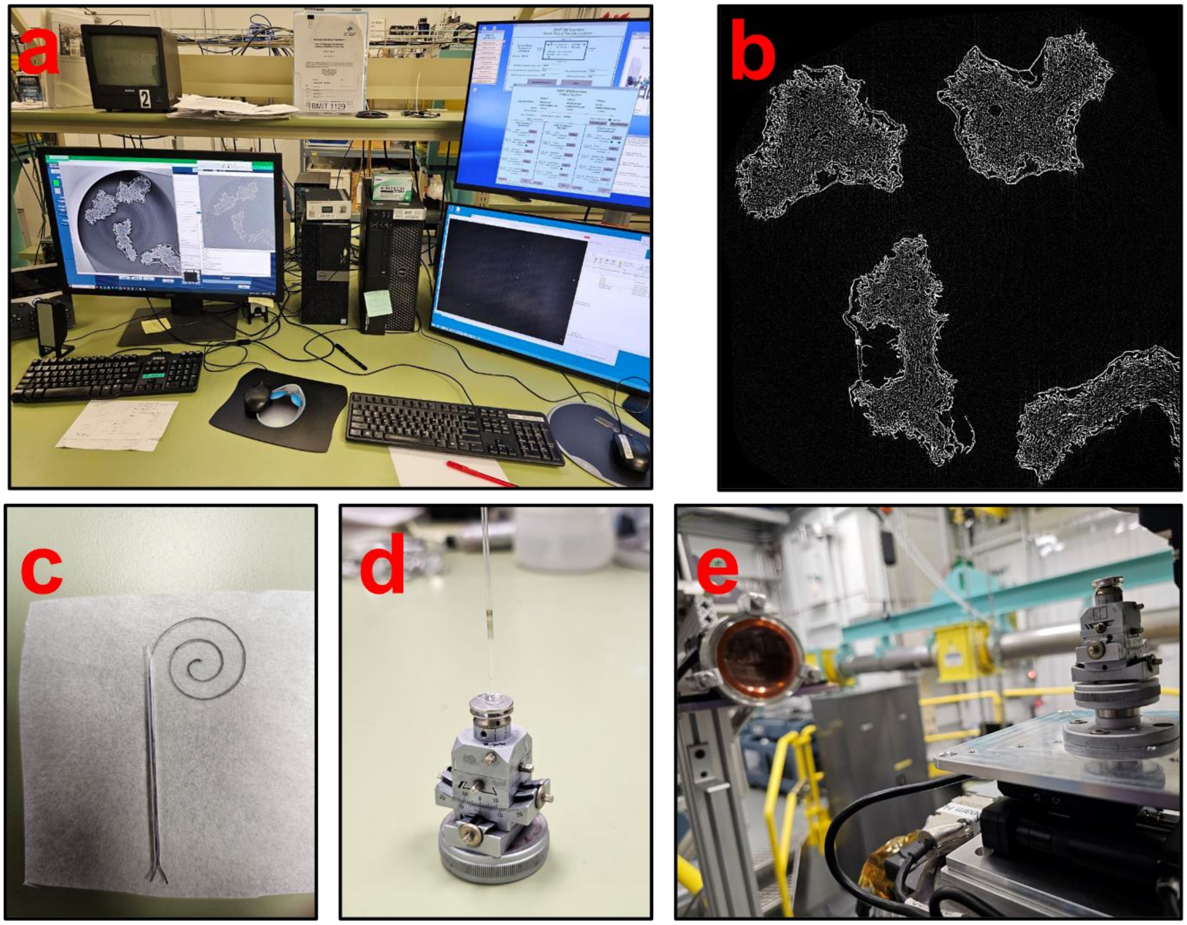

Figure S30. (a) Setup used for scanning and imaging of printed GO/sodium alginate filaments, showing the control systems and monitor displays, (b) processed 3D reconstructions of printed structures from scanning, (c) a printed spiral-shaped GO/sodium alginate filament sample, (d) filament sample mounted on the sample holder for scanning, and (e) setup of the sample holder within the beamline for high-resolution scanning.

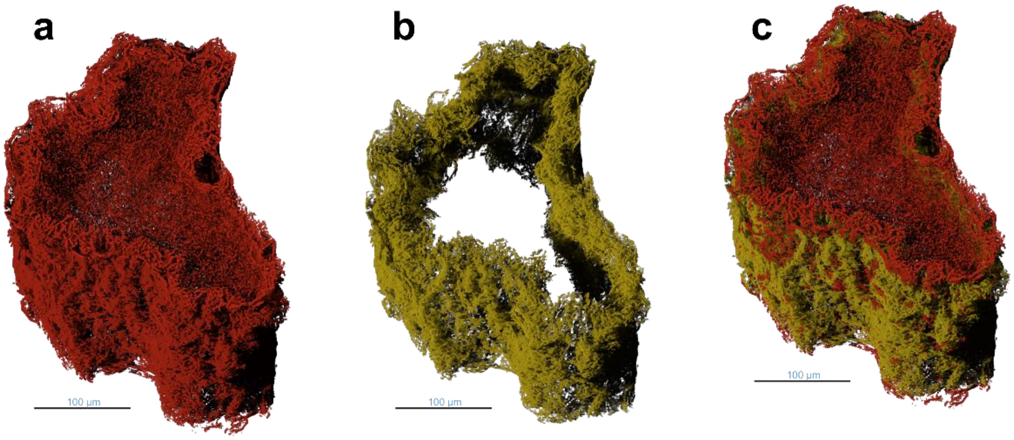

Figure S31. High-resolution 3D reconstructions of GO/sodium alginate filaments, showcasing the surface and internal structure with distinguishable skin-core configuration. The surface of the filament is shown in red (left), while the internal core structure is highlighted in yellow (middle), and the combined view (right) emphasizes the transition from core to surface. Scale bars represent 100 µm.

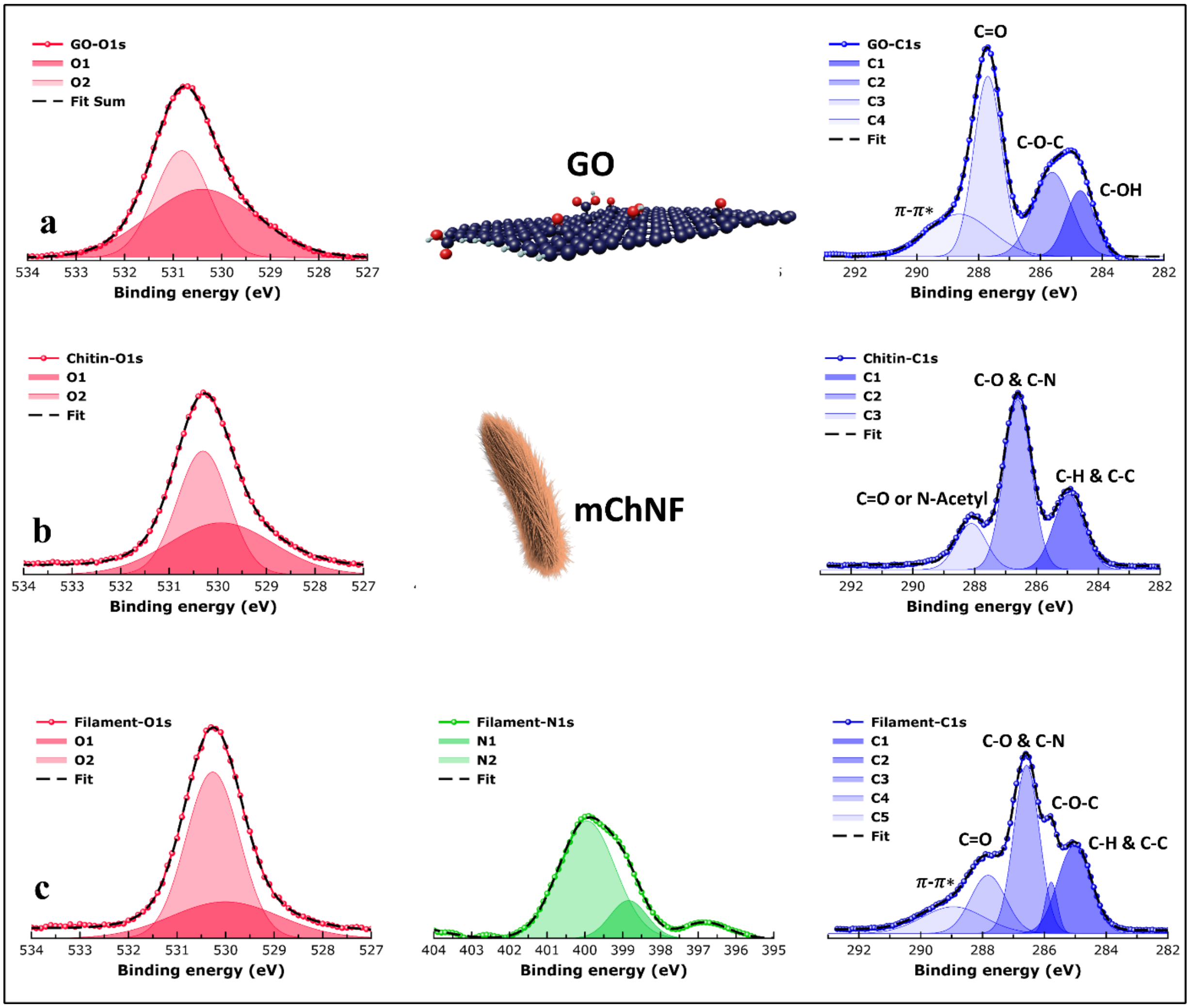

Figure S32. XPS analysis of GO and mChNF used to characterize their surface chemistry and bonding states. In the top row (a), the XPS spectra of GO show the deconvolution of O 1s and C 1s signals, indicating the presence of oxygen-containing functional groups such as C=O and C-OH. The molecular structure of GO is depicted in the center, showing the functionalized graphene structure. In the middle row (b), the XPS spectra of mChNF reveal the deconvolution of the O 1s and C 1s signals, highlighting the presence of various chemical states like C-O and C-N, typical of chitin and its modifications. A microstructure of the chitin nanofibers is illustrated in the center for visual reference. In the bottom row (c), the XPS spectra for the filament, display the O 1s, N 1s, and C 1s deconvolutions. This shows the characteristic binding energies for different functional groups, including C-O-C, C-H, and N-acetyl linkages, indicating successful integration of GO and mChNF.

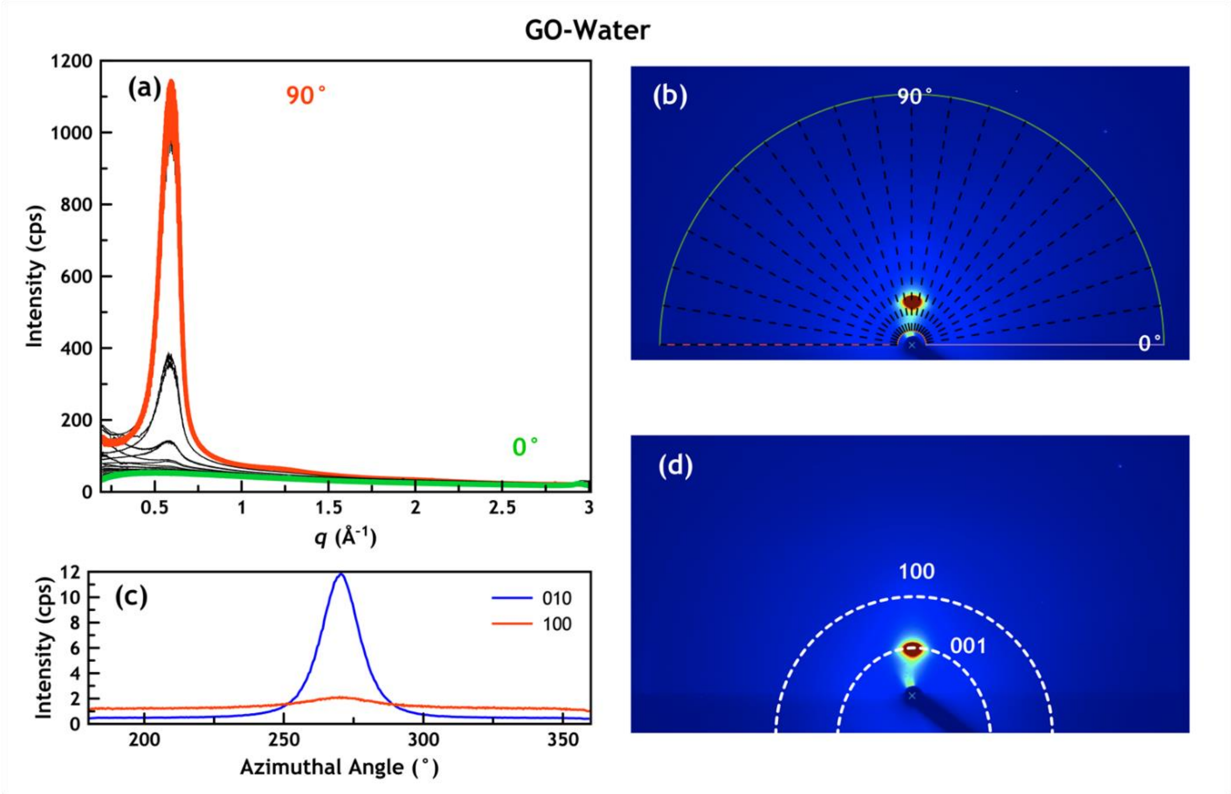

Figure S33. GIWAXS data for GO in a water matrix. (a) The GIWAXS intensity profiles at 0° and 90° display peaks corresponding to specific q-values, indicating different structural orientations of GO in water. (b) The GIWAXS pattern at 90° shows a distinct diffraction ring, signifying the alignment of GO sheets. (c) Azimuthal intensity plot for the (010) and (100) planes indicates anisotropic scattering, with peaks at specific azimuthal angles, suggesting preferred orientation. (d) The GIWAXS pattern highlights diffraction spots corresponding to the (001) and (100) planes, reflecting the crystalline structure of the GO in the water dispersion, dashed lines are representing the intensity plots on the signature Deby-Scherrer rings.

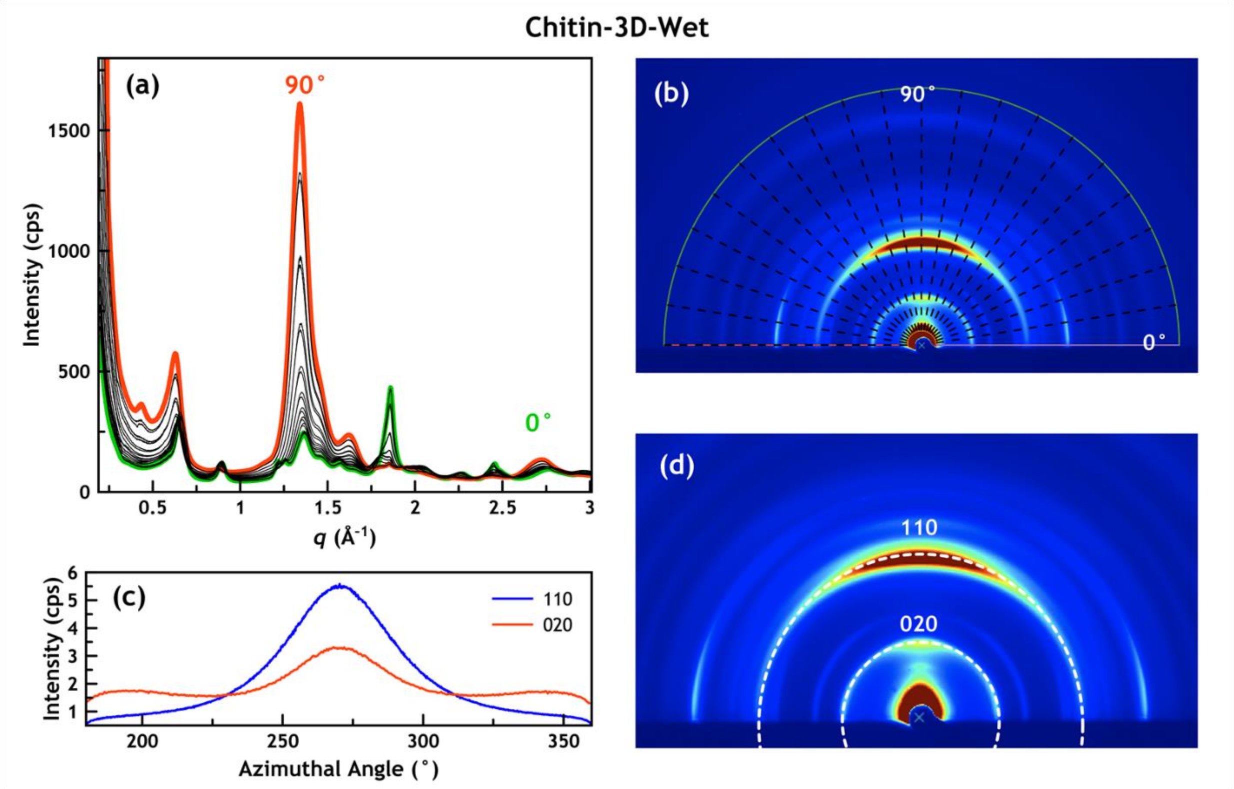

Figure S34. GIWAXS data for 3D chitin in a wet state. (a) The GIWAXS intensity profiles at 0° and 90° display peaks corresponding to specific q-values, indicating different structural orientations of chitin in its hydrated form. (b) The GIWAXS pattern at 90° shows distinct diffraction rings, signifying the crystalline alignment of chitin fibers. (c) Azimuthal intensity plot for the (110) and (020) planes indicates anisotropic scattering, with peaks at specific azimuthal angles, suggesting preferred fiber orientation. (d) The GIWAXS pattern highlights diffraction spots corresponding to the (110) and (020) planes, reflecting the crystalline structure of chitin, with dashed lines representing the intensity plots along Debye-Scherrer rings.

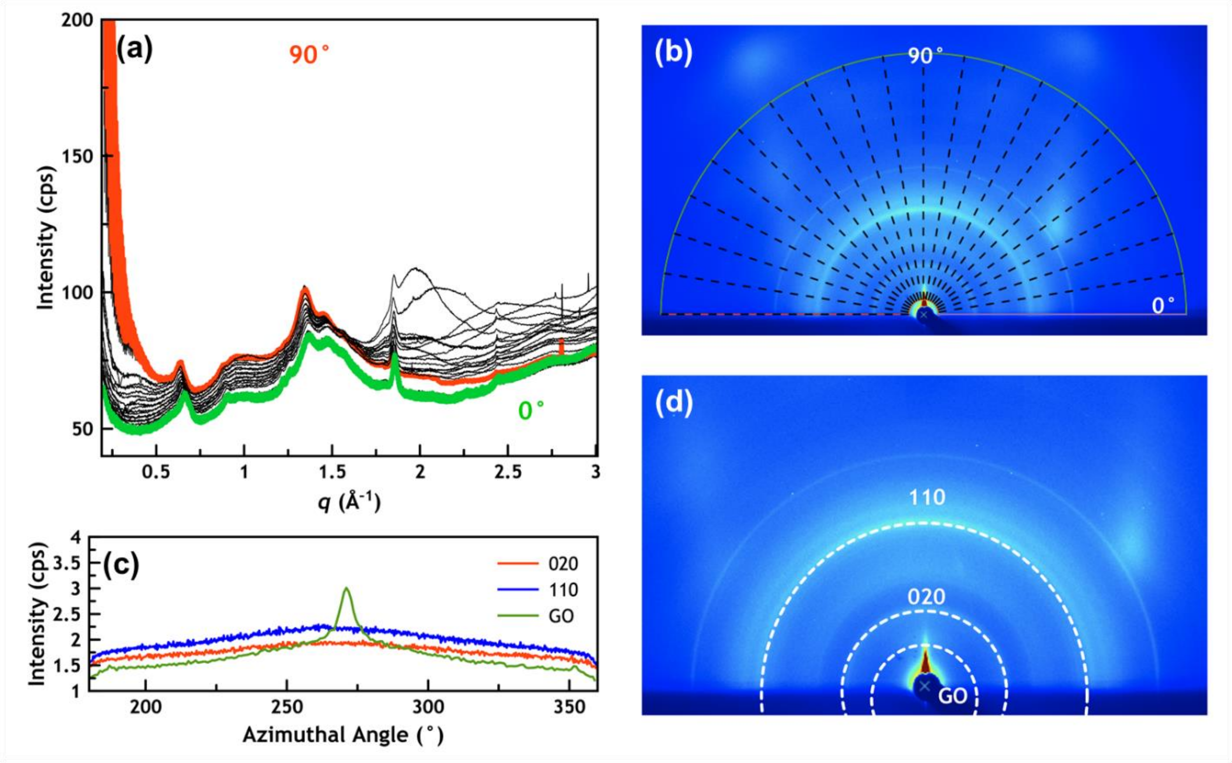

Figure S35. GIWAXS data for the filament sample. (a) The GIWAXS intensity profiles at 0° and 90° display peaks corresponding to specific q-values, indicating different structural orientations of the GO-chitin hybrid. (b) The GIWAXS pattern at 90° shows distinct diffraction rings, signifying the alignment of GO and chitin components within the hybrid material. (c) Azimuthal intensity plot for the (020), (110), and GO planes indicates anisotropic scattering, with peaks at specific azimuthal angles, suggesting preferred orientation of the hybrid structure. (d) The GIWAXS pattern highlights diffraction spots corresponding to the (110), (020), and GO planes, reflecting the crystalline structure of the GO-chitin hybrid, with dashed lines representing the intensity plots along Debye-Scherrer rings.

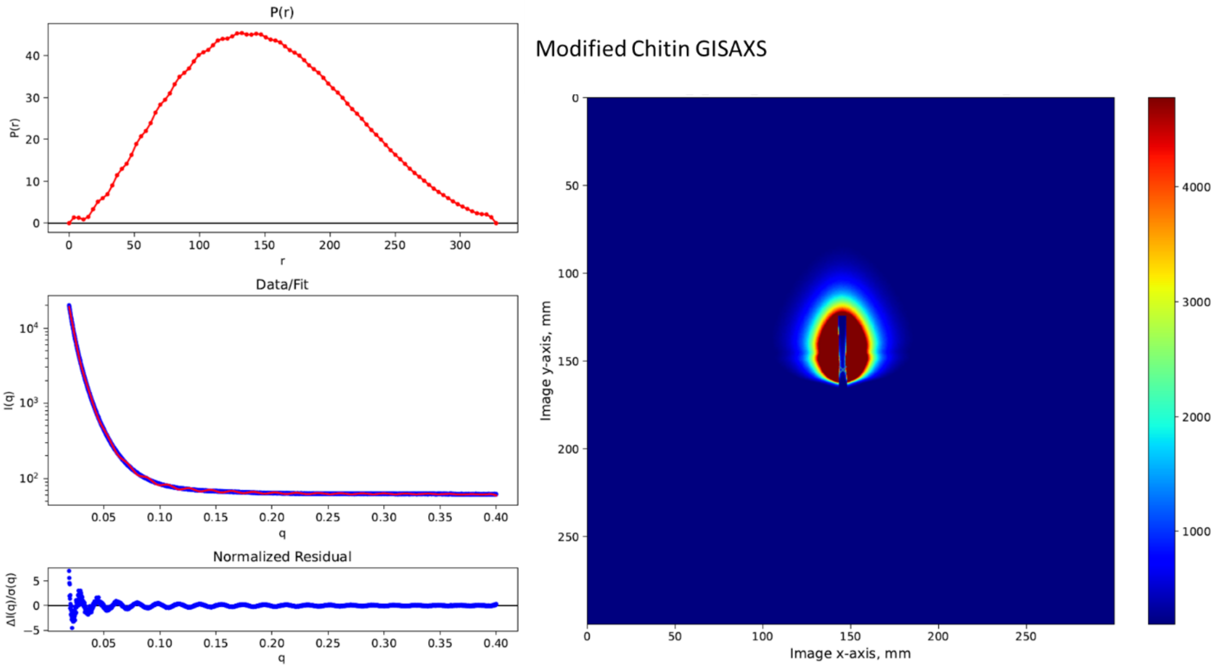

Figure S36. GISAXS data for modified chitin. (a) The pair distribution function (PDF) obtained from The Bayesian Inverse Fourier Transform (BIFT) analysis shows a broad peak, indicating the characteristic length scales of GO in the sample. (b) The distance distribution function, P(r), displays a typical decay profile, providing information on the particle size and distribution. (c) The normalized residuals oscillate around zero, indicating a good fit between the experimental data and the BIFT model. (d) The GISAXS pattern reveals anisotropic scattering, suggesting the alignment or orientation of GO layers, with intensity levels represented by the color scale on the right.

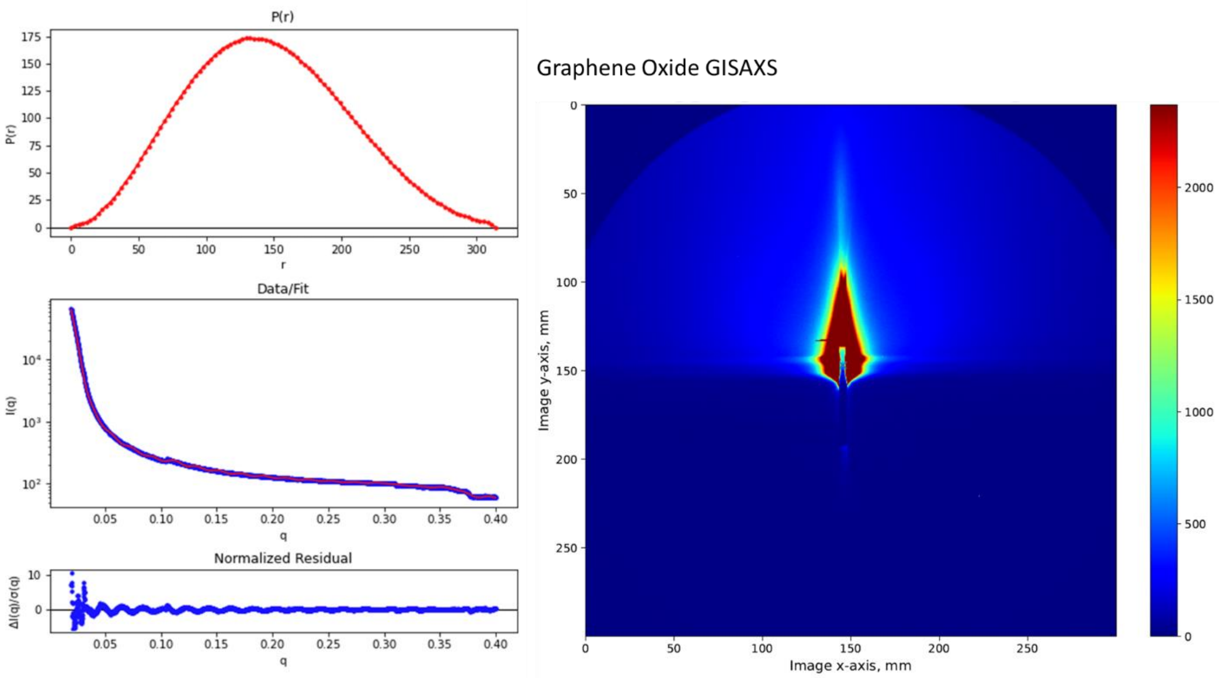

Figure S37. GISAXS data for GO. (a) The PDF derived from BIFT analysis displays a broad peak, indicating the characteristic length scales of the modified chitin in the sample. (b) The distance distribution function, P(r), exhibits a decay profile, consistent with the size and shape of the chitin particles. (c) The normalized residuals oscillate around zero, confirming a good fit of the model to the experimental data. (d) The GISAXS pattern reveals anisotropic scattering, suggesting alignment or orientation within the modified chitin fibers, with intensity levels represented by the color scale on the right.

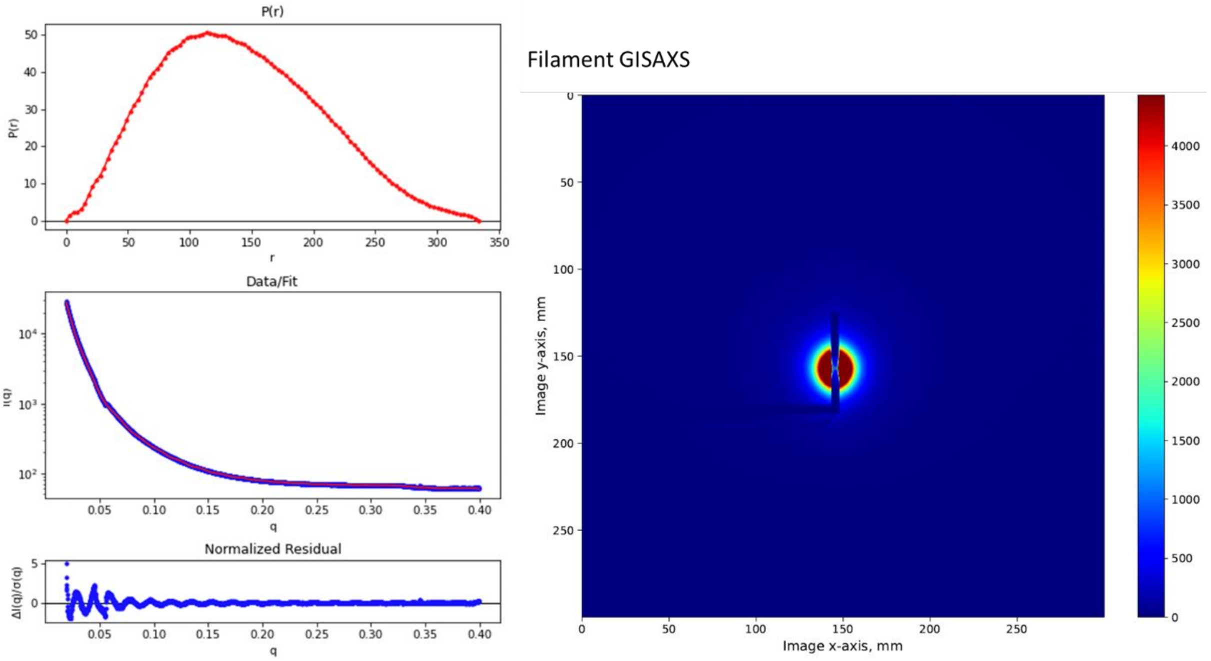

Figure S38. GISAXS data for the filament sample. (a) The PDF derived from BIFT analysis displays a broad peak, indicating the characteristic length scales within the filament sample. (b) The distance distribution function, P(r), shows a typical decay profile, providing insights into the particle size and structural arrangement in the filament. (c) The normalized residuals oscillate around zero, suggesting a good fit between the experimental data and the BIFT model. (d) The GISAXS pattern reveals isotropic scattering, indicating a uniform structure within the filament, with intensity levels represented by the color scale on the right.

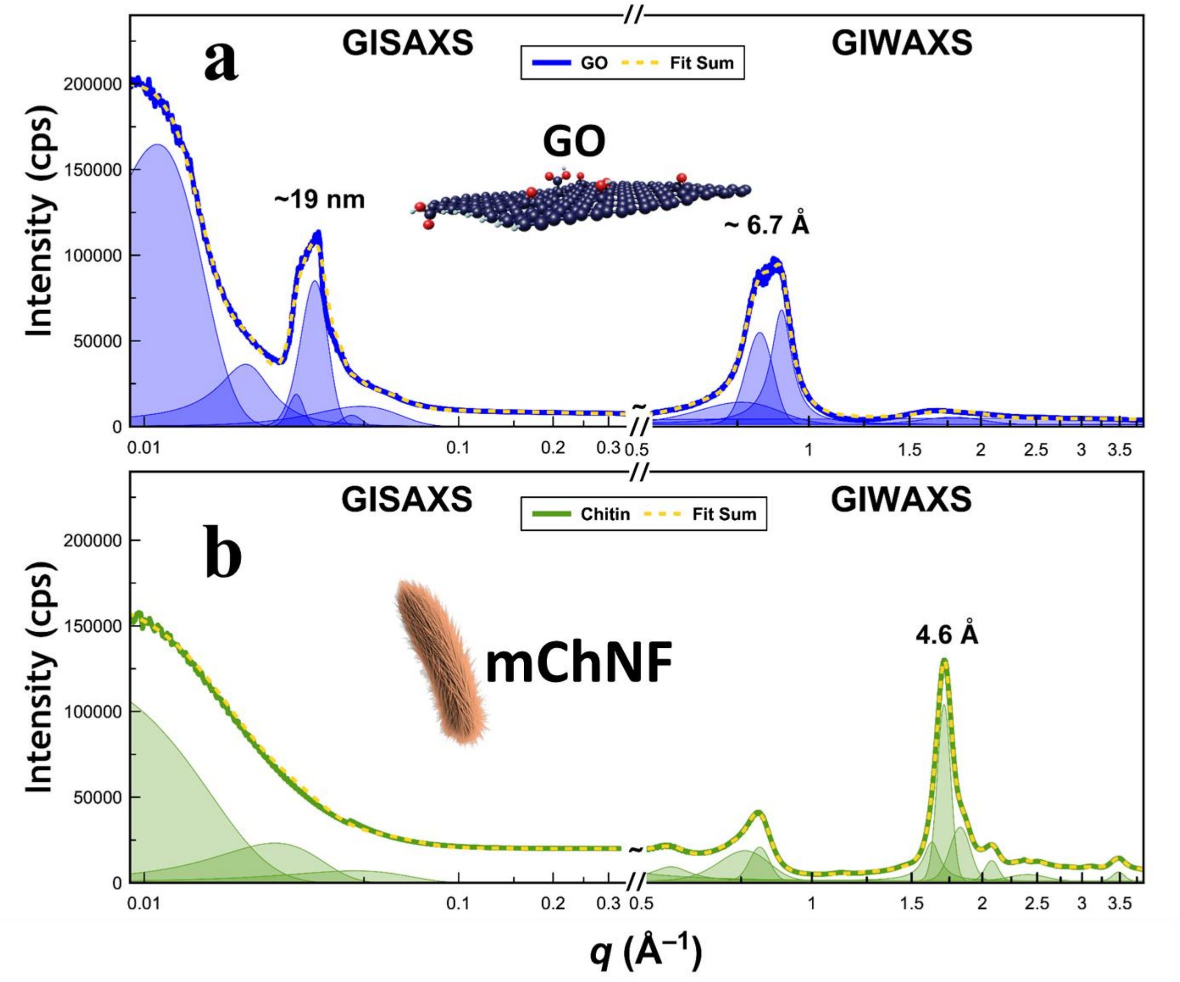

Figure S39. GISAXS and GIWAXS data for GO and mChNF samples. (a) The GISAXS and GIWAXS profiles for the GO sample exhibit characteristic peaks. The GISAXS region shows a broad peak corresponding to a particle size of ~19 nm, while the GIWAXS profile highlights a sharp peak at 6.7 Å, indicating the interlayer spacing within the GO sheets. The molecular structure of GO is illustrated between the profiles. (b) The GISAXS and GIWAXS profiles for the mChNF (modified chitin nanofiber) sample display characteristic peaks. The GISAXS region reveals scattering features indicative of the structural properties and packing of chitin, while the GIWAXS profile presents a peak at 4.6 Å, corresponding to the crystallographic spacing in the chitin structure. The structure of the mChNF is depicted between the profiles for clarity.

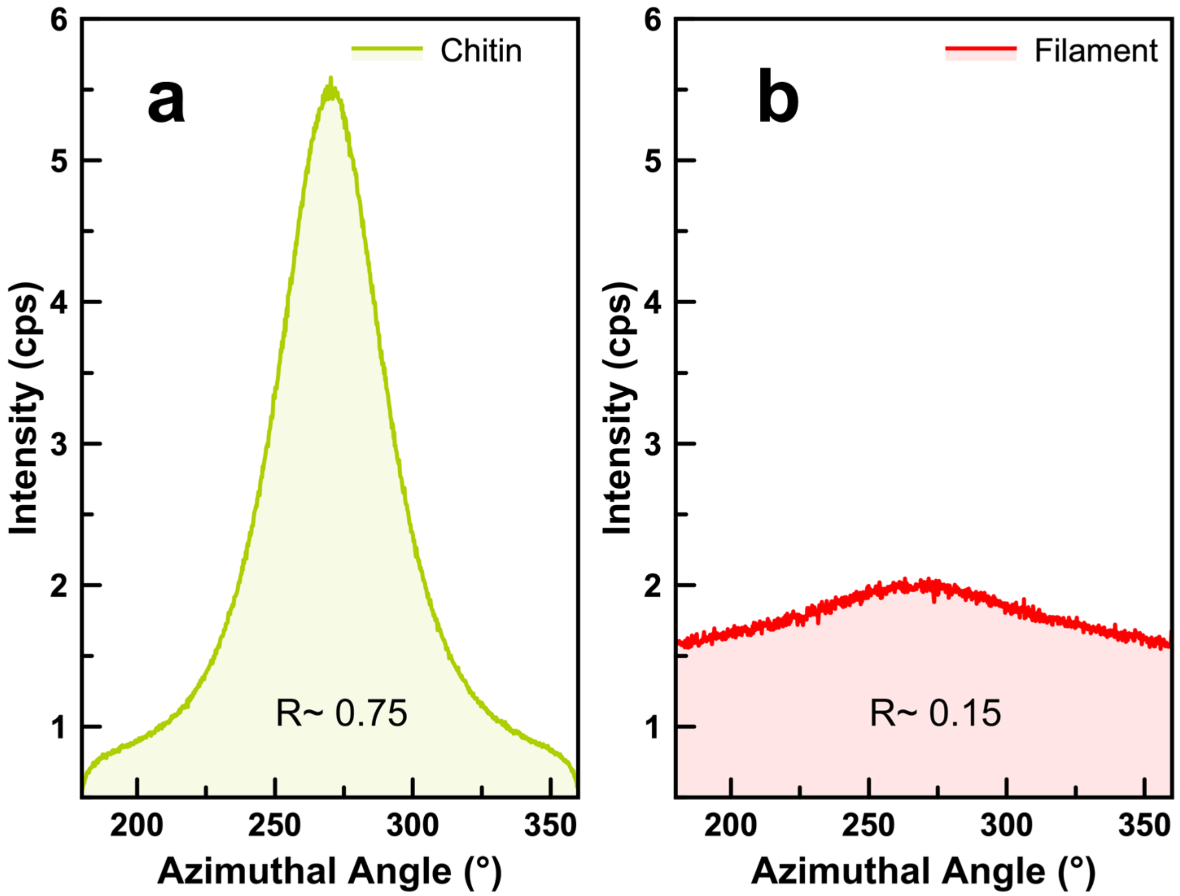

Figure S40. Azimuthal intensity plots for the Hermans Order Parameter analysis. (a) The plot for the cross-section mChNF film shows a pronounced peak with an azimuthal angle distribution at 270°, indicating a high degree of alignment. The Hermans Order Parameter (R) is calculated to be ~ 0.75, signifying a well-ordered structure in the chitin sample. (b) The plot for the filament sample shows a more uniform distribution of intensity across the azimuthal angles, with the Hermans Order Parameter (R) calculated to be ~ 0.15, indicating a lower degree of alignment in the filament structure. These plots reveal the comparative structural order in the chitin and filament samples, with chitin exhibiting a significantly higher alignment than the filament.

4. References

[1] D. C. Marcano, D. V. Kosynkin, J. M. Berlin, A. Sinitskii, Z. Sun, A. Slesarev, L. B. Alemany, W. Lu, J. M. Tour, *ACS Nano* **2010**, *4*, 4806.

[2] S. A. Hashemi, A. Ghaffarkhah, F. Ahmadijokani, H. Yousefian, S. E. Mhatre, A. Sinelshchikova, G. Banvillet, M. Kamkar, O. J. Rojas, S. Wuttke, M. Arjmand, *Nanoscale* **2024**, *16*, 8858.

[3] S. A. Hashemi, A. Ghaffarkhah, M. Goodarzi, A. Nazemi, G. Banvillet, A. S. Milani, M. Soroush, O. J. Rojas, S. Ramakrishna, S. Wuttke, T. P. Russell, M. Kamkar, M. Arjmand, *Advanced Materials* **2023**, *35*, DOI 10.1002/adma.202302826.

[4] A. Ghaffarkhah, S. A. Hashemi, F. Ahmadijokani, M. Goodarzi, H. Riazi, S. E. Mhatre, O. Zaremba, O. J. Rojas, M. Soroush, T. P. Russell, S. Wuttke, M. Kamkar, M. Arjmand, *Nat Commun* **2023**, *14*, DOI 10.1038/s41467-023-43319-7.

[5] F. Jiang, Y.-L. Hsieh, *Carbohydr Polym* **2013**, *95*, 32.

[6] Z. Zheng, H. L. Nguyen, N. Hanikel, K. K.-Y. Li, Z. Zhou, T. Ma, O. M. Yaghi, *Nat Protoc* **2023**, *18*, 136.

[7] B. H. Toby, R. B. Von Dreele, *J Appl Crystallogr* **2013**, *46*, 544.

[8] S. S. Nielsen, K. N. Toft, D. Snakenborg, M. G. Jeppesen, J. K. Jacobsen, B. Vestergaard, J. P. Kutter, L. Arleth, *J Appl Crystallogr* **2009**, *42*, 959.

[9] J. B. Hopkins, *J Appl Crystallogr* **2024**, *57*, 194.

[10] P. H. Hermans, J. J. Hermans, D. Vermaas, *Journal of Polymer Science* **1946**, *1*, 162.

[11] O. Kose, C. E. Boott, W. Y. Hamad, M. J. MacLachlan, *Macromolecules* **2019**, *52*, 5317.

[12] N. Fairley, V. Fernandez, M. Richard-Plouet, C. Guillot-Deudon, J. Walton, E. Smith, D. Flahaut, M. Greiner, M. Biesinger, S. Tougaard, D. Morgan, J. Baltrusaitis, *Applied Surface Science Advances* **2021**, *5*, 100112.

[13] O. BETZ, U. WEGST, D. WEIDE, M. HEETHOFF, L. HELFEN, W. LEE, P. CLOETENS, *J Microsc* **2007**, *227*, 51.

[14] P. Cloetens, W. Ludwig, J. Baruchel, D. Van Dyck, J. Van Landuyt, J. P. Guigay, M. Schlenker, *Appl Phys Lett* **1999**, *75*, 2912.

[15] J. B. Hopkins, R. E. Gillilan, S. Skou, *J Appl Crystallogr* **2017**, *50*, 1545.

[16] X. Xiao, X. Huang, A. Wang, S. Cao, M. Noroozi, M. Panahi-Sarmad, *Carbohydr Polym* **2022**, *281*, 119042.

[17] M. Panahi-Sarmad, E. Chehrazi, M. Noroozi, M. Raef, M. Razzaghi-Kashani, M. A. Haghighat Baian, *ACS Appl Electron Mater* **2019**, *1*, 198.

[18] M. Panahi-Sarmad, M. Abrisham, M. Noroozi, V. Goodarzi, M. Arjmand, M. Sadri, P. Dehghan, A. Amirkiai, H. A. Khonakdar, *Eur Polym J* **2020**, *133*, 109745.

[19] M. Panahi-Sarmad, V. Goodarzi, A. Amirkiai, M. Noroozi, M. Abrisham, P. Dehghan, Y. Shakeri, N. Karimpour-Motlagh, F. Poudineh Hajipoor, H. Ali Khonakdar, A. Asefnejad, *Eur Polym J* **2019**, *118*, 619.

[20] D. López-Díaz, M. López Holgado, J. L. García-Fierro, M. M. Velázquez, *The Journal of Physical Chemistry C* **2017**, *121*, 20489.

[21] O. V. Tomchuk, M. V. Avdeev, A. T. Dideikin, A. Ya. Vul', A. E. Aleksenskii, D. A. Kirilenko, O. I. Ivankov, D. V. Soloviov, A. I. Kuklin, V. M. Garamus, Yu. V. Kulvelis, V. L. Aksenov, L. A. Bulavin, *Diam Relat Mater* **2020**, *103*, 107670.

[22] W. H. Ferreira, K. Dahmouche, C. T. Andrade, *Carbohydr Polym* **2019**, *208*, 124.

[23] Y. Lei, I. Boucenna, V. Thévenet, W. Brett, E. Paineau, A. Ponton, *Langmuir* **2024**, *40*, 16048.

[24] G. I. Titelman, V. Gelman, S. Bron, R. L. Khalfin, Y. Cohen, H. Bianco-Peled, *Carbon N Y* **2005**, *43*, 641.

[25] S. Sakurai, *Polym Int* **2017**, *66*, 237.

[26] P. Fratzl, *J Appl Crystallogr* **2003**, *36*, 397.

[27] Y. B. Melnichenko, G. D. Wignall, *J Appl Phys* **2007**, *102*, DOI 10.1063/1.2759200.

[28] V. Gerold, G. Kostorz, *J Appl Crystallogr* **1978**, *11*, 376.

[29] M. Roman, W. T. Winter, *Biomacromolecules* **2004**, *5*, 1671.

[30] W. Ruland, *Colloid Polym Sci* **1977**, *255*, 417.

[31] J. S. Pedersen, P. Schurtenberger, *Macromolecules* **1996**, *29*, 7602.
